# Uncovering Aggregation‐Induced Emission in Carbon Dots for Color‐Changing Hydrogels and Information Encryption

**DOI:** 10.1002/anie.7943243

**Published:** 2026-06-13

**Authors:** Jiafeng Wan, Shiyi Chen, Haopu Su, Tim Muschik, Tongtong Cui, Yosuke Akae, Dominik Voll, Christian W. Schmitt, Stefan Bräse, Tao Chen, Andreas‐Neil Unterreiner, Patrick Théato

**Affiliations:** ^1^ Institute For Chemical Technology and Polymer Chemistry (ITCP) Karlsruhe Institute of Technology (KIT) Karlsruhe Germany; ^2^ Institute of Organic Chemistry (IOC) Karlsruhe Institute of Technology (KIT) Karlsruhe Germany; ^3^ Institute of Physical Chemistry (IPC) Karlsruhe Institute of Technology (KIT) Karlsruhe Germany; ^4^ Research Fellow of Japan Society For the Promotion of Science Tokyo Japan; ^5^ Soft Matter Synthesis Laboratory – Institute For Biological Interfaces III (IBG‐3) Karlsruhe Institute of Technology (KIT) Eggenstein‐Leopoldshafen Germany; ^6^ Institute of Biological and Chemical Systems – Functional Molecular Systems (IBCS‐FMS) Karlsruhe Institute of Technology (KIT) Karlsruhe Germany; ^7^ State Key Laboratory of Advanced Marine Materials Zhejiang Key Laboratory of Extreme‐environmental Material Surfaces and Interfaces Ningbo Institute of Materials Technology and Engineering Chinese Academy of Sciences Ningbo China; ^8^ School of Chemical Sciences University of Chinese Academy of Sciences Beijing China

**Keywords:** aggregation‐induced emission (AIE), anticounterfeiting printing, carbon dots, fluorescent hydrogels, information encoding

## Abstract

Since the discovery of carbon dots (CDs), the typical precursor combination of citric acid (CA)‐urea has been widely used in the scientific community to explore the formation mechanism and luminescence behavior of CDs. However, there have only been a few reports on the synthesis of CDs featuring aggregation‐induced emission (AIE) characteristics. In this study, CA and urea were used to synthesize hydrophilic red‐emissive carbon dots (R‐CDs) that exhibit blue fluorescence in water (dispersed state) and red fluorescence in DMF (aggregated state). The study reveals that the photoluminescence of R‐CDs is governed by π–π stacking interactions between solute molecules as well as solvent effects between solute and solvent molecules, leading to solvent‐responsive emission behavior. By tuning the solvent polarity, the intermolecular distance between R‐CDs can be adjusted, thereby influencing their photoluminescent properties. Taking advantage of the solvent‐responsive color change, anticounterfeiting printing and information encryption applications were designed. Moreover, by combining R‐CDs with poly(vinyl alcohol) (PVA), hydrogel‐based fluorescent information‐encoding materials were successfully fabricated.

## Graphical abstract

Red‐emissive carbon dots (R‐CDs) exhibiting aggregation‐induced emission (AIE) and aggregation‐caused quenching (ACQ) effects are synthesized from citric acid and urea. They display weak blue fluorescence in water but strong red emission in DMF due to aggregation. By tuning π–π stacking and solvent interactions, tunable solvent‐responsive fluorescence is achieved, enabling advanced applications in information encryption and smart luminescent hydrogels.

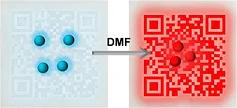

## 1 Introduction

Carbon dots (CDs) are zero‐dimensional carbon‐based nanomaterials with sizes typically below 10 nm [1, 2], and were first incidentally discovered by Xu et al. [3]. in 2004 during the preparation of carbon nanotubes. Subsequently, in 2006, the group of Sun successfully synthesized CDs via laser ablation and formally coined the term “carbon dots” [4]. CDs have attracted tremendous research interest because of their outstanding optical properties, which include their tunable photoluminescence, high fluorescence intensity, quantum yield, and remarkable resistance to photobleaching, as well as their excellent biocompatibility, low toxicity, abundant precursor availability, and cost‐effective preparation [5, 6, 7, 8, 9, 10]. They have been widely explored for applications in bioimaging [11], drug delivery [12], catalysis [13], sensors [14], light‐emitting diodes (LEDs) [15, 16], soft robotics [17, 18], and information encryption [19].

For most of the CDs reported to date, a pronounced aggregation‐caused quenching (ACQ) effect is observed. Specifically, these CDs exhibit strong fluorescence in the dispersed state, whereas their emission is readily quenched upon aggregation [20]. In 2001, the group of Tang [21] discovered that hexaphenylsilole (HPS) was nonemissive in solution but displayed intense fluorescence in the aggregated state, and for the first time proposed the concept of aggregation‐induced emission (AIE). AIE is defined as a phenomenon in which molecules are non‐ or weakly emissive in a solubilized or dispersed state but become strongly emissive in an aggregated or solid state, owing to the restriction of intramolecular motion that suppresses nonradiative decay pathways [22]. At the early stage of its discovery, AIE‐related studies were primarily focusing on organic fluorescent dyes [23].

In 2013, Gao et al. [24] synthesized blue‐emissive carbon quantum dots (CQDs) with a high photoluminescence quantum yield of up to 80% from C_60_, and, for the first time, reported their aggregation‐induced emission (AIE) behavior. Since then, the concept of AIE has been progressively introduced into the research field of CDs [25], and in recent years, CDs featuring AIE behavior have gradually received increasing attention and have been extensively studied. In 2019, Yang et al. [26] designed N,S‐doped hydrophobic CDs (H‐CD) using dithiosalicylic acid (DTSA) and melamine (MA) as precursors. These H‐CDs exhibited blue emission in the dispersed state but showed red emission in the aggregated state, thus demonstrating remarkable AIE properties. Moreover, reversible dual‐switching luminescence was realized through the disulfide bonds in DTSA. Based on this seminal work, subsequent studies employed DTSA to successfully synthesize various types of AIE‐active CDs with tunable emission colors [27, 28, 29, 30]. For instance, Zhang et al. [31] reported the preparation of hydrophobic solid‐state red fluorescent hydrophobic carbon nanodots (M‐CDs) from DTSA and manganese acetate, and further incorporated them into agar hydrogels, preparing a solvent‐responsive, color‐switchable fluorescent hydrogel.

With the in‐depth exploration of AIE CDs, researchers have recently identified additional precursors that can also be used to synthesize AIE CDs and have further investigated the underlying mechanisms. Ding et al. [32] used thiosalicylic acid and carbonyl hydrazine as precursors, and successfully synthesized blue, green, yellow, and red AIE CDs by changing the reaction temperature and the precursor ratio. Their study revealed that the red‐shift of the emission wavelength originated from the extension of the sp^2^ conjugated domains as well as the incorporation of graphitic nitrogen. In another study, Ji et al. [33] demonstrated that hydrogen‐bond‐mediated self‐assembly constitutes the fundamental mechanism of cluster‐induced emission in CDs, thereby enabling precise modulation between particle emission and cluster emission.

The citric acid (CA)‐urea system, a classical model for synthesis of CDs, has long been regarded as an important subject for investigating the synthetic process, photoluminescence mechanisms, and related theoretical aspects [34, 35]. Qu et al. [36] reported the solvothermal synthesis of orange‐emissive CDs from CA and urea, and their results demonstrated that DMF serves as an effective solvent for promoting the formation of large conjugated sp^2^ domains. In a previous study, the CA‐urea system was successfully used to prepare a full color range of CDs, including blue, green, yellow, orange, and red emissions [37]. Subsequently, there has been a report describing the presence of concentration‐dependent luminescence in CDs derived from the CA‐urea system [38]. At high concentrations, the intermolecular forces led to a reduced distance betweenCDs, facilitating the interaction of surface functional groups such as carbonyl and amino groups. This narrowed the energy bandgap, resulting in a red‐shift of the emission. Conversely, at low concentrations, the increased interparticle distance weakened these interactions, leading to a blue‐shift in fluorescence.

**SCHEME 1:**
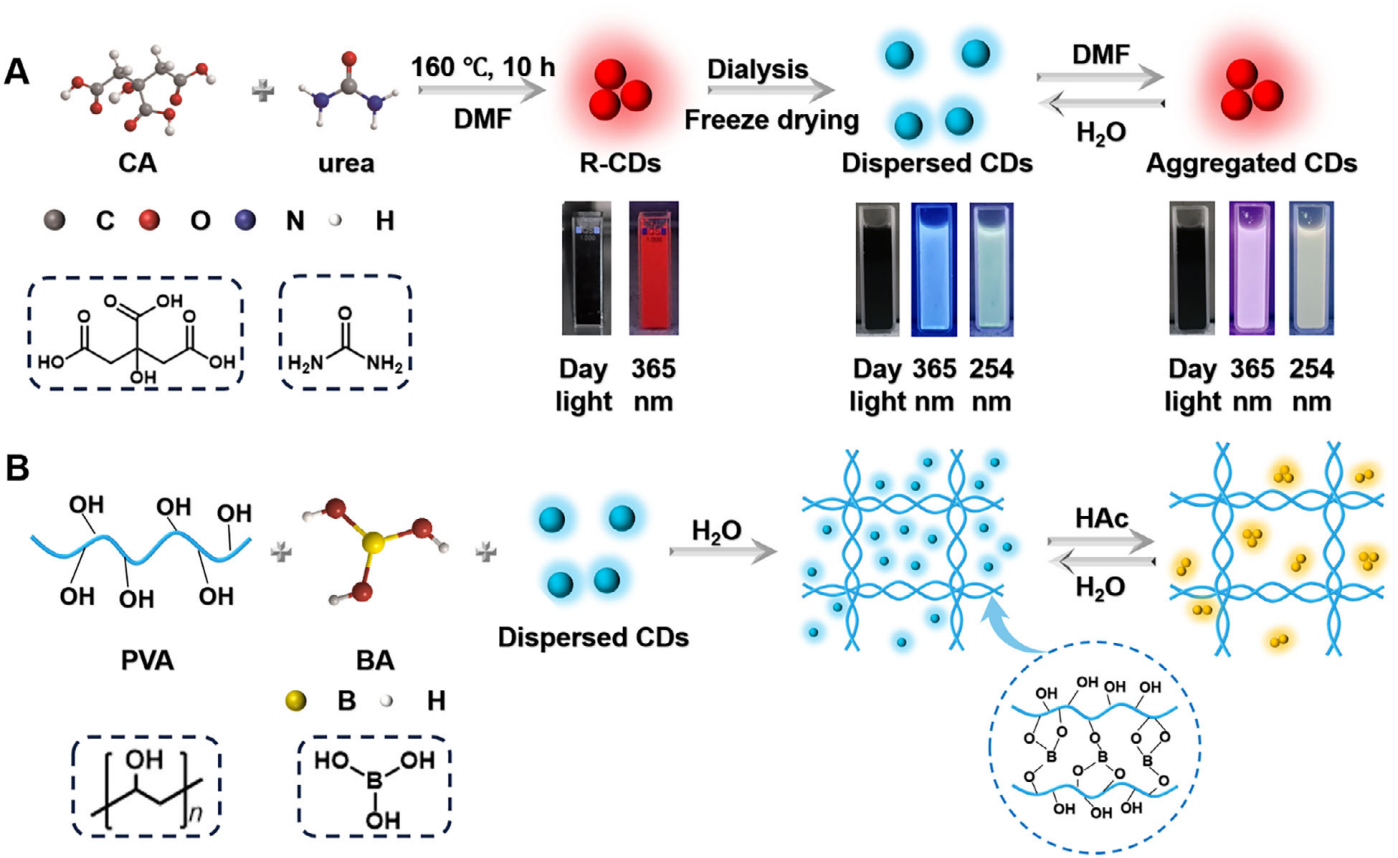
(A) The preparation of R‐CDs; (B) The preparation of PVA‐based fluorescent hydrogels.

Building upon our previous study, we herein describe the synthesis of hydrophilic AIE red‐emissive carbon dots (R‐CDs) via a solvothermal method using DMF as the solvent and CA‐urea as precursors. The primary objective of this study is to explore the potential AIE properties within the CA‐urea system, filling the gap regarding its underexplored solvent‐responsive luminescent behavior. We aim to demonstrate, via NMR characterization, that the introduction of DMF facilitates the formation of extended conjugated structures, endowing R‐CDs with the unique capability to dynamically switch between blue fluorescence in aqueous dispersion and red emission in the aggregated state. Furthermore, we systematically investigate the photoluminescence mechanisms and the underlying ultrafast dynamics using femtosecond transient absorption spectroscopy and steady‐state optical measurements. Finally, taking advantage of the solvent‐responsive nature of R‐CDs, we target the development of multifunctional platforms for information security and smart fluorescent hydrogels (Scheme 1B).

## 2 Results and Discussion

### 2.1 Preparation and Morphological Characterizations of R‐CDs

Red‐emissive carbon dots (R‐CDs) were synthesized via a solvothermal reaction of CA and urea in DMF at 160°C for 10 h following a recent study [37]. After filtration, dialysis, and freeze‐drying, the final R‐CDs were obtained in a 12.7% yield. In this synthesis, DMF not only functioned as the reaction medium but also actively participated in the synthesis process, contributing additional nitrogen to the formation of the CDs. When dispersed in water, the R‐CDs exhibited weak blue fluorescence upon UV irradiation at 365 nm, whereas the addition of DMF resulted in the observation of a bright red emission (refer to Scheme 1A). Importantly, the dialysis purification procedure played a critical role in determining the photophysical behavior of the products. During dialysis, water molecules gradually diffused into the dialysis bag, and with extended dialysis time, the R‐CDs became uniformly dispersed in the aqueous phase. Consequently, the freeze‐dried R‐CDs obtained after dialysis were significantly more loosely packed. It is worthy to note that R‐CDs isolated by solvent precipitation followed by high‐speed centrifugation did not exhibit such distinct phenomenon of fluorescence color changes [37].

**FIGURE 1:**
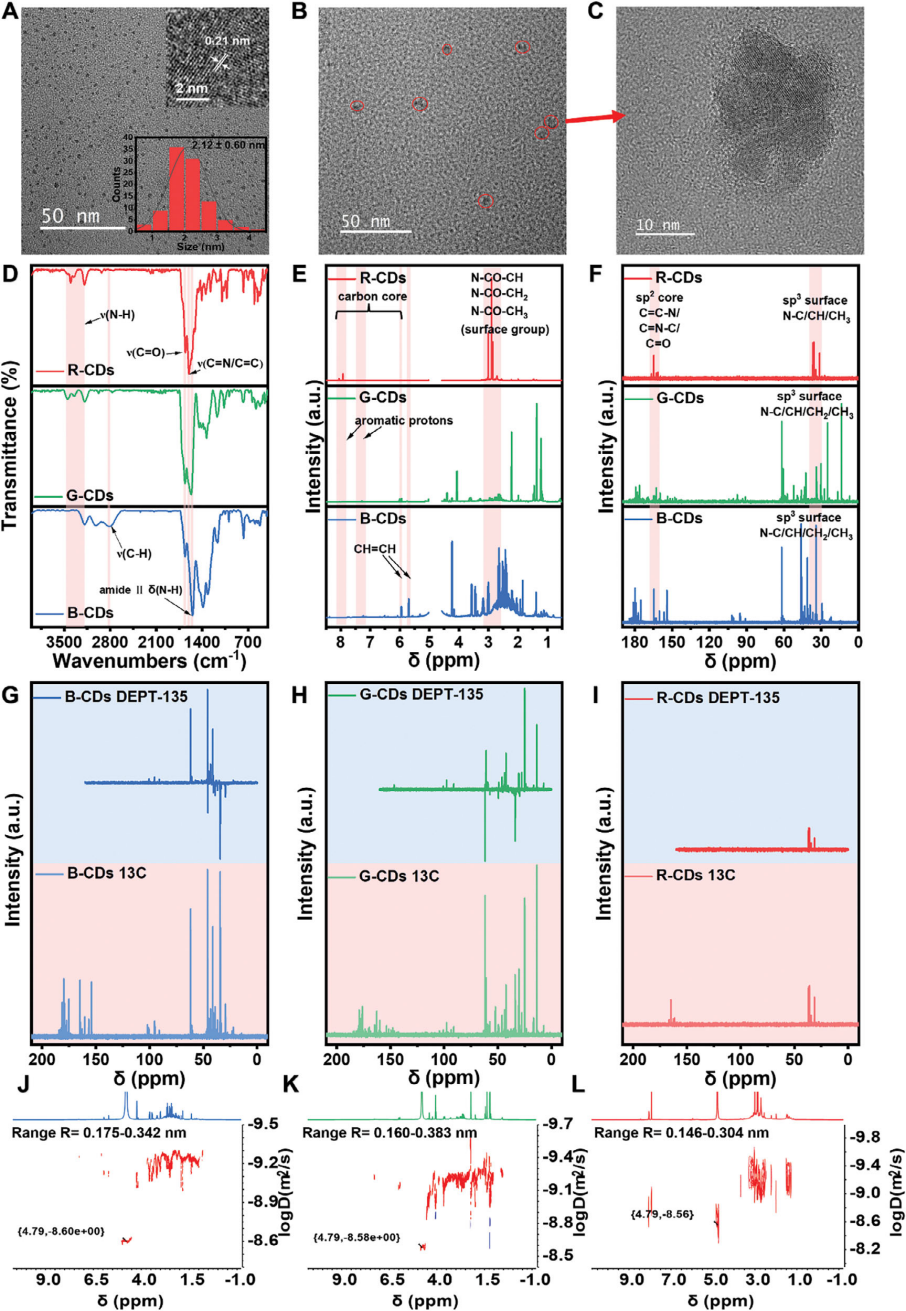
(A) TEM image of R‐CDs (water as the dispersant). The image in the upper right corner is an HRTEM (High Resolution Transmission Electron Microscopy) picture, and the image in the lower right corner shows the size of R‐CDs; (B) and (C) TEM imagine of R‐CDs (ethanol as the dispersant); (D) FTIR spectra of multicolor CDs; (E) ^1^H NMR spectra of multicolor CDs (solvent: D_2_O, 500 MHz, 25°C); (F) ^13^C NMR spectra of multicolor CDs (solvent: D_2_O, 500 MHz, 25°C); (G), (H) and (I) are DEPT‐135 NMR spectra of multicolor CDs; (J), (K) and (L) are DOSY NMR spectra of multicolor CDs (solvent: D_2_O, 500 MHz, 25°C).

The obtained R‐CDs were uniformly dispersed, with an average particle size of approximately 2.12 nm (refer to Figure 1A). High‐resolution TEM (HRTEM) images revealed a lattice spacing of about 0.21 nm, which corresponds to the (100) plane of graphitic carbon, indicating that the R‐CDs possess a certain degree of graphitization [39]. Ethanol was used as a dispersant to further observe the dispersion behavior of R‐CDs (refer to Figure 1B,C). The R‐CDs tended to aggregate in ethanol, forming larger nanoparticle clusters as observed by TEM.

### 2.2 Structural Characterizations of Multicolor CDs

To explain the reason why R‐CDs exhibited an aggregation behavior in DMF, blue‐emissive carbon dots (B‐CDs) and green‐emissive carbon dots (G‐CDs) were synthesized first by using water and ethanol as solvents respectively, under identical reaction conditions (see Figure S1).

To investigate the structural characteristics of the obtained CDs, FTIR, and NMR analyses were performed for the CDs synthesized in these different solvents. As shown in Figure 1D, B‐CDs exhibited a distinct band at 1550 cm^−^^1^, which was attributed to the N─H bending vibration of the ─CO─NH─ formed through the dehydration and condensation between CA and urea [40]. In the case of G‐CDs, this band slightly shifted toward higher wavenumbers, suggesting that a portion of the amide groups participated in intramolecular cyclization reactions, resulting in a change in their chemical environment. For R‐CDs, the sharp band around 1600 cm^−^^1^ mainly corresponded to the stretching vibrations of aromatic C═C and C═N bonds. In water, CDs formed under milder conditions, yielding relatively simple carbon‐core structures that retained substantial functional groups from the precursors. Consequently, B‐CDs exhibited broad bands within the 1100–1400 cm^−^^1^ range, alongside aliphatic C─H stretching vibrations at 2800 cm^−^^1^. Ethanol is a moderately polar solvent, in which many functional groups of the precursors were consumed, forming a partial aromatic structure. Therefore, a relatively dense surface functional group appeared in the range of 1100 –1400 cm^−1^, while the C─H stretching vibration band of the aliphatic group at 2800 cm^−1^ disappeared, and a stronger signal of an aromatic N─H bond (3200–3400 cm^−1^) was formed. DMF, as a strong polar, nonprotonic solvent, promoted cyclization and aromatization reactions of CDs. The involvement of nitrogen in DMF additionally facilitated the formation of conjugated structures such as pyridine and pyrrole units leading to larger conjugated π systems. This explains why R‐CDs exhibited complex band patterns within the 1100–1400 cm^−^^1^ range.

These structural evolutions were further corroborated by the ^1^H NMR spectra. As depicted in Figure 1E, the upfield region corresponded to the aliphatic protons attached to the sp^3^‐hybridized carbons on the CDs' surface [26]. Compared to B‐CDs and G‐CDs, R‐CDs exhibited sparse yet highly resolved resonances in this region, implying a structurally conjugated framework with a highly homogeneous chemical environment. Conversely, the downfield region revealed the aromatic proton signals of the carbon core. For B‐CDs, the resonances observed at ∼5.76 and ∼6.0 ppm were assigned to short‐chain or isolated olefinic protons formed during the initial synthesis stage. As carbonization proceeds, G‐CDs exhibited aromatic proton signals at 7.3 ppm, indicating the gradual formation of cyclized structures and an elevated degree of conjugation. R‐CDs displayed prominent highly conjugated aromatic proton peaks at 7.9 and 8.0 ppm, explicitly demonstrating the formation of a massive sp^2^‐conjugated carbon core, which served as one of fundamental driving force for their red‐shifted emission [32, 41].

The ^13^C NMR spectra provided further validation for the conjugated evolution (depicted in Figure 1F). For R‐CDs, the signals in both sp^2^ aromatic region and sp^3^ aliphatic region were fewer but concentrated, reiterating their ordered structure. Notably, the reduction of prominent resonances in the 170–180 ppm range suggested a significant depletion of carboxyl groups (─COOH), caused by the deep dehydration and carbonization processed that concomitantly facilitate the expansion of the conjugated aromatic domains. More conclusively, the DEPT‐135 NMR spectra revealed the presence of ─CH_2_ groups in both B‐CDs and G‐CDs, whereas such signals were entirely absent in R‐CDs (see Figure 1G, H, I). It was well‐established that within an extensively conjugated aromatic network, carbon atoms predominantly exist in the form of ─CH and terminal ─CH_3_, intrinsically precluding the existence of ─CH_2_ groups. This unambiguous absence of─CH_2_ definitively confirmed that R‐CDs possessed a vast and continuous conjugated structural core with minimal residual aliphatic chains.

The hydrodynamic radius for the three CDs was calculated from DOSY NMR results: R‐CDs (0.146–0.304 nm), G‐CDs (0.160–0.383 nm), and B‐CDs (0.175–0.342 nm) (shown in Figure 1J, K,L). These results indicate that, from B‐CDs to G‐CDs and finally to R‐CDs, the molecular size first increased and then decreased. The formation process of CDs can be described as the initial polymerization of small molecules into polymeric chains, followed by cross‐linking to form preliminary CDs structures. As the reaction proceeded, dehydration and carbonization occurred, leading to a more regulated structure [37].

**FIGURE 2:**
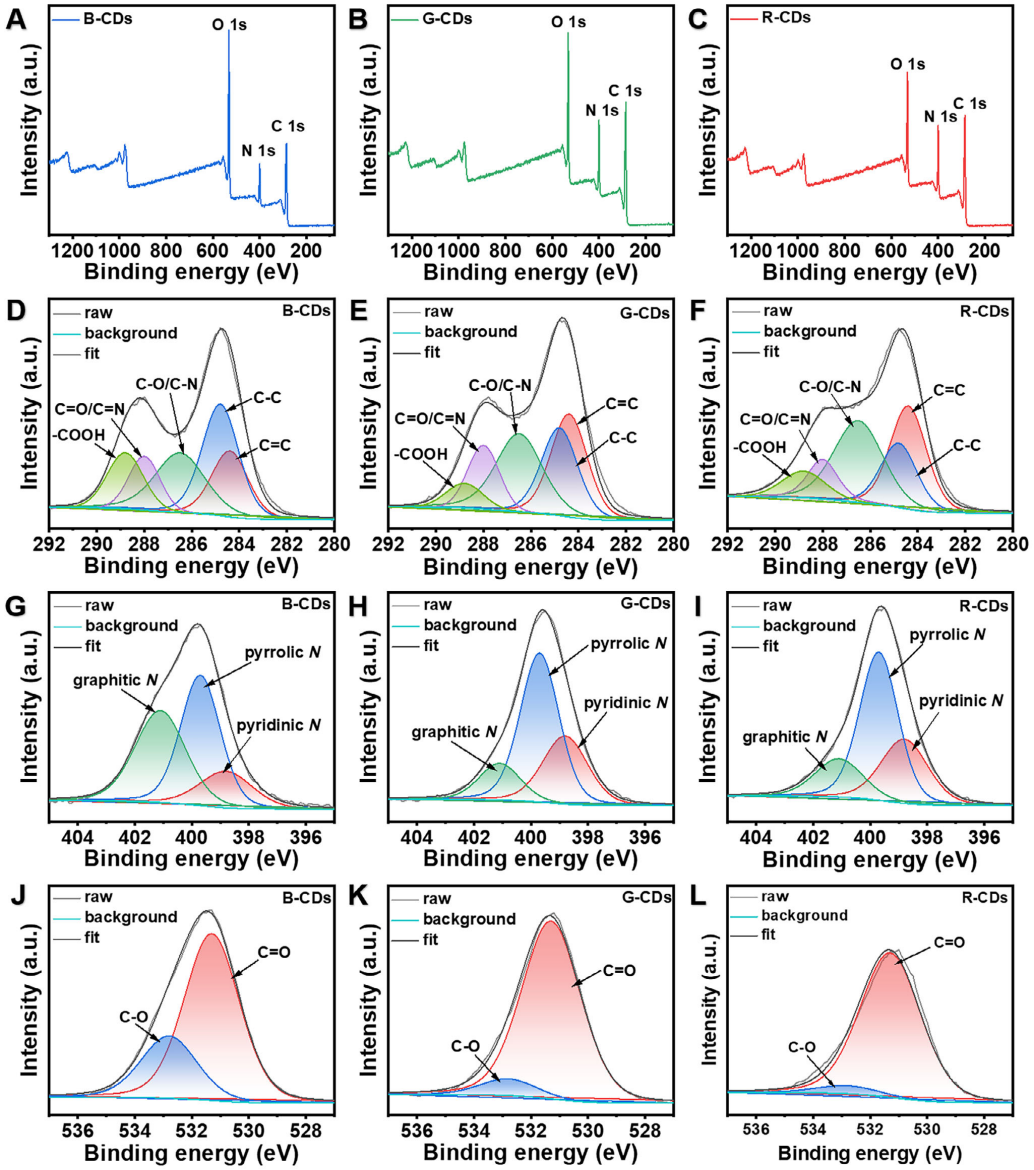
High‐resolution XPS survey spectra of (A) B‐CDs, (B) G‐CDs and (C) R‐CDs; high‐resolution XPS C 1s spectra of (D) B‐CDs, (E) G‐CDs and (F) R‐CDs; high‐resolution XPS N 1s spectra of (G) B‐CDs, (H) G‐CDs and (I) R‐CDs; high‐resolution XPS O 1s spectra of (J) B‐CDs, (K) G‐CDs and (L) R‐CDs.

Figure 2 A, B,C present the XPS survey spectra of B‐CDs, G‐CDs, and R‐CDs, respectively. Table S1 summarizes the elemental compositions (C, N, and O) of multicolor CDs. As shown in the Table S1, R‐CDs exhibited a higher nitrogen content and a lower oxygen content compared to B‐CDs and G‐CDs. This suggested that DMF, acting as a solvent, participated in the reaction, thereby enhancing the nitrogen doping of R‐CDs while promoting a higher degree of carbonization and dehydration. Analysis of the high‐resolution C 1s spectra revealed that for R‐CDs, the content of C═C (284.4 eV) increased while that of C─C (284.8 eV) decreased [42, 43], providing evidence for the enhanced conjugation in R‐CDs (refer to Figure 2D, E, F and Table S2). Furthermore, while the C─O/C─N (286.4 eV) content increased [44], the O 1s spectra (as shown in Figure 2J,K,L) and Table S4 indicated a significant reduction in C─O (532.8 eV) for R‐CDs [37], implying a higher content of C─N bonds within the structure. B‐CDs possessed a substantial amount of ─COOH (288.8 eV) (refer to Table S2). This is attributed to the relatively mild synthesis conditions in water, where the aqueous environment inhibited the processes of carbonization and dehydration. In contrast, in an ethanol environment, the hydroxyl groups of ethanol underwent esterification with CA or the carboxyl groups on the CD surface. This formed a termination layer around CDs, inhibiting further growth and carbonization of the carbon core. Consequently, the C 1s spectrum of G‐CDs showed a higher C═O/C═N (288.0 eV) content and lower ─COOH content (refer to Table S2). For R‐CDs, although the C═O/C═N content in the C 1s spectrum was lower (refer to Table S2), the C═O (531.3 eV) content in the O 1s spectrum was the highest among the samples (refer to Table S4). This indicated that the DMF environment facilitated deep dehydration and carbonization, leading to the formation of more conjugated C═O bonds. Regarding N 1s spectra (Figure 2G,H,I), R‐CDs exhibited high proportions of pyridinic ***N*** (398.8 eV, 28.51%) and pyrrolic ***N*** (399.7 eV, 54.17%) (refer to Table S3). Notably, the graphitic ***N*** (401.1 eV) content reached 17.32% [37], further contributing to the large conjugated structure of R‐CDs. It is worth mentioning that although B‐CDs showed a graphitic N content of 41.36%, the XPS C 1s and NMR spectra suggested the absence of a large conjugated domain. Therefore, it was highly probable that B‐CDs contained significant amounts of protonated amine groups (e.g., ─NH_3_^+^), whose peaks overlapped with that of graphitic N, resulting in an apparent broad peak [45, 46].

Combining the FTIR and NMR analyses, it can be concluded that when water was used as solvent, the reaction between CA and urea tended to stop at the early stage of polycondensation, yielding polymeric intermediates rich in carboxyl and hydroxyl groups. These species exhibited low degrees of dehydration and carbonization, resulting in small‐sized carbon cores with limited sp^2^ conjugation domains and abundant oxygen‐containing surface groups (─COOH, ─OH). Consequently, B‐CDs remained in a high‐energy state, emitting blue fluorescence. When ethanol was used as the solvent, condensation and amidation between CA and urea occurred concurrently with esterification reactions involving ethanol. The resulting G‐CDs possessed moderately extended sp^2^ conjugation domains, while their surfaces were passivated by ester and amide species, preventing excessive carbonization. This led to a more red‐shifted emission than B‐CDs, producing a green fluorescence instead. In contrast, when DMF was used as the solvent, its thermal decomposition at high temperature and pressure generated dimethylamine and formic acid, which participate in the synthesis of R‐CDs. These intermediates introduced different nitrogen configurations (graphitic ***N***, pyridinic ***N***, and pyrrolic ***N***), promoting aromatization and graphitization. As a result, R‐CDs formed larger sp^2^‐conjugated domains responsible for their low‐energy red emission centers.

### 2.3 Characterization of the Optical Properties of R‐CDs

**FIGURE 3:**
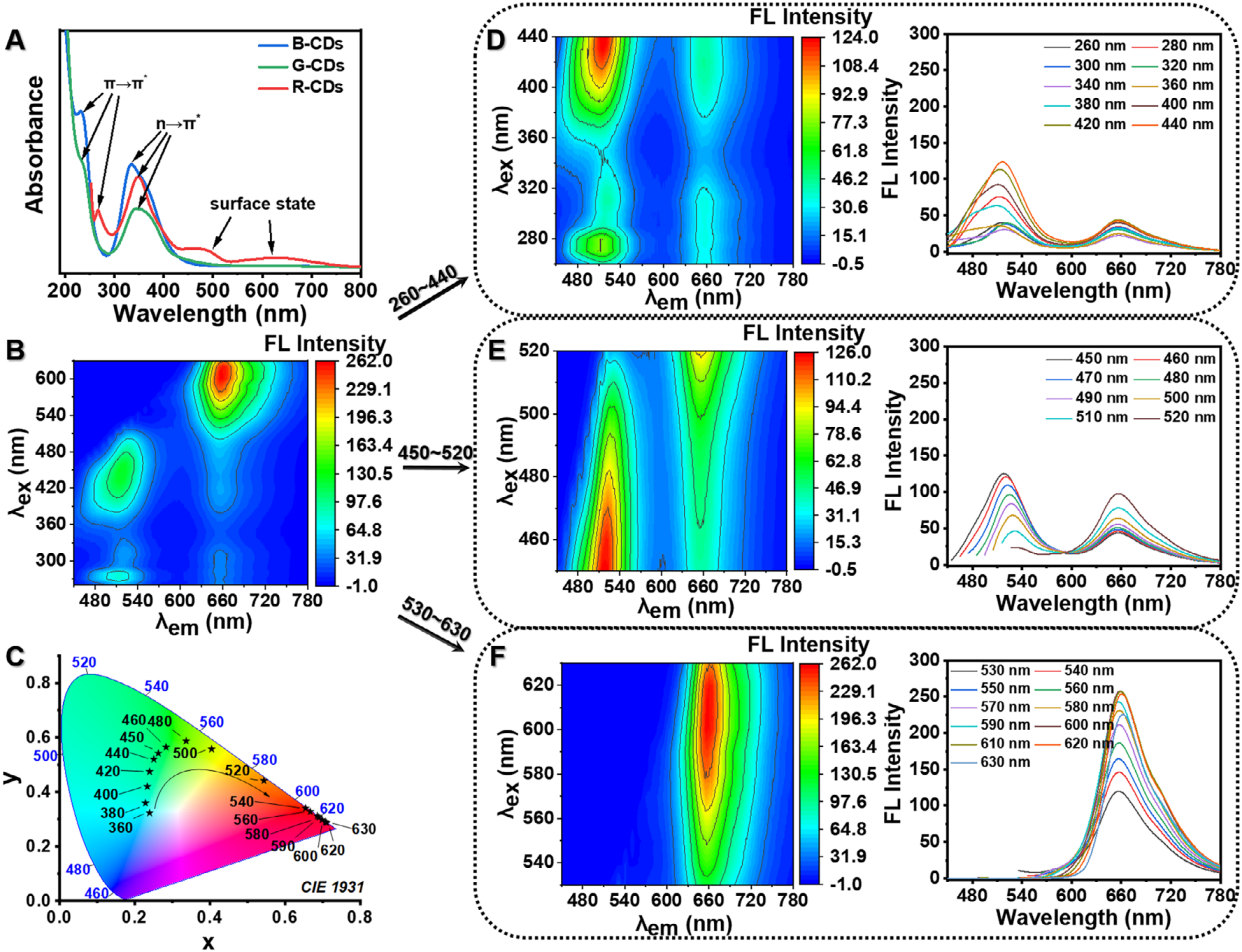
Characterizations of the optical properties of R‐CDs: (A) UV–vis spectra of B‐CDs, G‐CDs, and R‐CDs; (B) FL spectrum of R‐CDs; (C) chromaticity diagram of R‐CDs; (D) FL spectra of R‐CDs (excitation wavelength: 260–440 nm); (E) FL spectra of R‐CDs (excitation wavelength: 450–520 nm); (F) FL spectra of R‐CDs (excitation wavelength: 530–630 nm).

Figure 3A depicts the UV–vis spectrum of B‐CDs, G‐CDs and R‐CDs. The absorption peaks observed in the 230– 260 nm range are attributed to π → π* electronic transitions, originating from the C═C/C═N bonds within the sp^2^‐hybridized carbon network [47, 48]. According to the quantum confinement effect, an expanded conjugation system enhanced π‐electron delocalization, thereby reducing the energy gap between the HOMO and LUMO and decreasing the energy required for transitions. Consequently, both absorption and emission wavelengths underwent a redshift [49, 50]. As illustrated in the spectra, π → π* transition peak of R‐CDs exhibited a significant redshift, providing strong evidence that R‐CDs possessed a larger conjugated domain compared to B‐CDs and G‐CDs. The peaks appearing between 330 and 350 nm corresponded to n → π* nonelectronic transitions, which stemmed from surface‐state C═O and C═N [51]. The expansion of the conjugated domain led to a lowering of the π* energy level [52], while the increase in conjugated C═O and aromatic nitrogen (e.g., pyridinic ***N***) elevated the n energy level [53]. This combined effect resulted in the observed redshift of the n → π* transition peak for R‐CDs. Furthermore, R‐CDs exhibited multiple absorption peaks in the 400–700 nm range, indicating their ability to undergo electronic transitions at lower excitation energy and emit red fluorescence. These peaks likely originated from a “push‐pull” electron system constructed between pyrrolic N (donor) and C═O or pyridinic N (acceptor) moieties [29]. In summary, the large conjugated structure of R‐CDs facilitated the occurrence of π‐π stacking interactions in solution.

Through fluorescence (FL) spectroscopy, the photoluminescent behavior of R‐CDs in DMF was analyzed (refer to Figure 3B). Two emission centers were observed, with the first emission center located around an emission wavelength of 520 nm, and its optimal excitation wavelength at about 450 nm. Another emission center was located around an emission wavelength of 660 nm, and its optimal excitation wavelength was about 600 nm. The fluorescence spectrum was divided into three sections based on the excitation wavelength: 1) From 260 to 440 nm, two stable emission centers (emission wavelength: 520 and 660 nm) were observed (see Figure 3D); 2) From 450 to 520 nm, there were also two groups of emission centers in this range (shown in Figure 3E). With the increase of excitation wavelength, the emission of the high‐emission‐wavelength emission center on the right remained stable around 660 nm, while the emission of the low‐emission‐wavelength emission center on the left continuously shifted to longer wavelengths, and the fluorescence intensity gradually decreased until it extinguished. 3) From 530 to 630 nm, there was only one group of emission centers (refer to Figure 3F), which remained stable at 660 nm. This emission center exhibited extremely strong fluorescence intensity.

Based on the FL spectrum of R‐CDs, its chromaticity diagram was drawn (see Figure 3C). As shown, the black stars represent the excitation wavelengths of R‐CDs. As the excitation wavelength increased, the fluorescence color of R‐CDs gradually changed from blue to red.

### 2.4 The Cause of Red Fluorescence

To investigate the origin of the red fluorescence of R‐CDs, four different CDs were synthesized. As shown in Figure S2, CDs‐1 (blue fluorescence) and CDs‐2 (yellow fluorescence) were synthesized by using CA or urea alone under the same reaction conditions. Then, CA was first reacted in a hydrothermal reactor for 5 h, and the resulting blue fluorescent solution was further reacted with urea via a solvothermal reaction for 5 h to obtain CDs‐3 (greenish‐yellow fluorescence). Finally, CA was reacted first by hydrothermal reaction for 5 h, then filtered, dialyzed to remove unreacted CA molecules, and freeze dried. The purified CA CDs powder was then subjected to a solvent‐thermal reaction with urea for 10 h to obtain CDs‐4 (yellow fluorescence).

Figure S3 shows their corresponding FL spectra, while Figure S3A shows the FL spectrum of CDs‐1. Based on the photoluminescence behavior, the FL spectrum was divided into two parts (excitation wavelengths: 260–430 nm and 440–600 nm) (refer to Figure S3B,C). In Figure S3B, a stable emission center with two adjacent emission peaks at 470 and 490 nm was observed, which resulted in a blue fluorescence. When the excitation wavelength reached 440 nm, the two adjacent peaks merged into a single emission peak. As the excitation wavelength increased, the emission center shifted toward the higher wavelength region. When the excitation wavelength exceeded 550 nm, a stable emission center peak appeared around 640 nm (depicted in Figure S3C). Similarly, the FL spectrum of CDs‐2 (shown in Figure S3D) was also divided into two parts based on excitation wavelength and photoluminescence behavior (260–490 nm and 500–600 nm) (refer to Figure S3 E,F). These two parts exhibited similar photoluminescence behavior: a stable emission center (emission wavelength: 546 nm) and an emission center that shifted with the excitation wavelength. The FL spectra of CDs‐3 and CDs‐4 also exhibited this similar photoluminescence behavior (shown in Figure S3 G, L).

From Figure S3 A,D, it can be seen that CA can provide blue emission, while urea can provide orange emission. Figure S4 A–D shows a mixture of CDs‐1 and CDs‐2 in equal volumes and its FL spectra. Comparing the FL spectra of the mixture with that of R‐CDs (see Figure S4 E,F), their FL spectra were different. Therefore, the photoluminescence behavior of R‐CDs was not a simple superposition of photoluminescence behavior of CDs‐1 and CDs‐2. This indicated that only the interaction between CA and urea could produce red fluorescent CDs. Independent CA and urea could not produce red fluorescence under the same conditions. Compared to our previous studies, it was found that CDs synthesized by water as a solvent resulted in only a stable set of emission centers. However, in this work, CDs synthesized by DMF as a solvent exhibited not only stable emission centers but also emission centers with excitation‐dependent fluorescence. This was due to DMF participating in the reaction during CDs synthesis, leading to the formation of complex functional groups on the CDs surface. The doping of nitrogen (***N***) originating from DMF facilitated the formation of graphitic ***N***, pyridinic ***N***, and pyrrolic ***N***, which in turn promoted the formation of conjugated π systems [37]. The increased delocalization of π electrons narrowed the energy gap between the HOMO and LUMO, resulting in a red shift of the CDs fluorescence emission wavelength [32, 36]. Additionally, the introduction of DMF caused CDs system (CA and urea) to form an intrinsic state of the carbon core (emission center at 520 nm) and surface state fluorescence (emission center at 660 nm).

### 2.5 Aggregation‐Induced Emission of R‐CDs

The reversible color change of the R‐CD solution provided a vivid macroscopic illustration of AIE. As shown in Video S1, a dilute aqueous solution of R‐CDs exhibited only a weak blue fluorescence under 365 nm UV illumination. The addition of DMF switched the emission to a bright pink color, while the re‐addition of deionized water restored the original blue fluorescence, and a second DMF addition again produced the pink emission. This behavior reflects the formation of π‐π stacked aggregates in the less polar solvent (DMF) environment, which gave rise to an additional, red shifted fluorescence band that was absent when R‐CDs were well dispersed in water. Moreover, this solvent‐responsive emission demonstrated good reversibility, which can be well‐maintained throughout three alternating DMF/water treatment cycles (see Figure S5).

The FL spectra of R‐CDs before and after color change were tested separately. In Figure S6, the FL spectra of R‐CDs in water is shown. It can be seen that R‐CDs exhibited weak blue fluorescence in water, with emission wavelengths varying with excitation wavelengths, demonstrating excitation dependence. Upon addition of DMF, R‐CDs exhibited strong red fluorescence, with photoluminescence behavior consistent with that in pure DMF.

**FIGURE 4:**
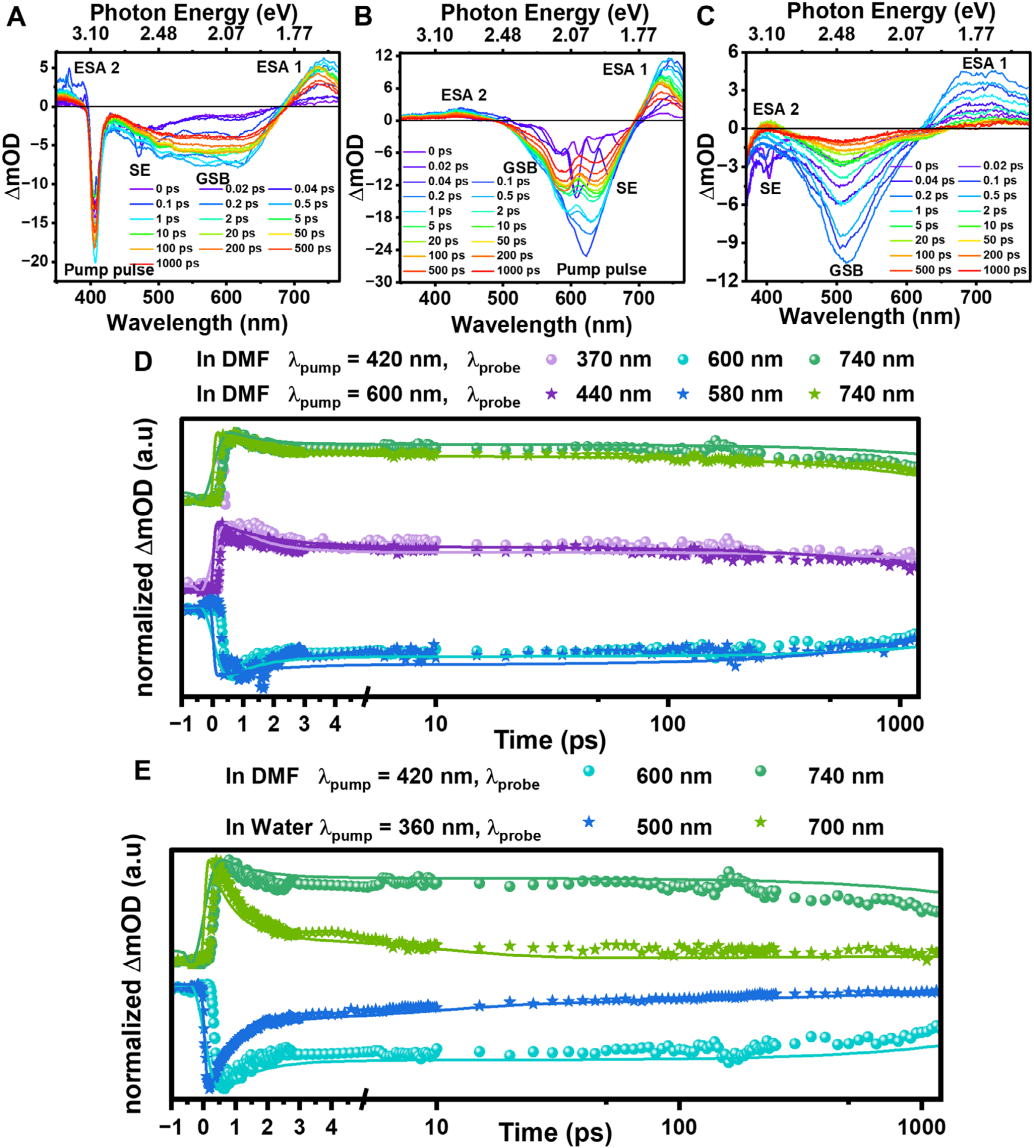
Transient absorption spectra for R‑CDs. (A) DMF as solvent, excitation at 420 nm (OD  =  0.81; E_pulse_  =  0.30 µJ). (B) DMF as solvent, excitation at 600 nm (OD  =  1.42; E_pulse_  =  0.30 µJ). (C) Water as solvent, excitation at 360 nm (OD  =  1.10; E_pulse_  =  0.30 µJ). (D) Normalized single‑transient kinetics for the DMF samples (420 nm and 600 nm excitation) together with the global‑analysis fit (colored lines). (E) Normalized single‑transient kinetics for the water sample (360 nm excitation) together with the global‑analysis fit (colored lines).

In DMF the probe windows were chosen to avoid overlap with the pump spectral tails (370 nm/600 nm for 420 nm excitation; 440 nm/580 nm for 600 nm excitation) (shown in Figure 4A,B). Under these conditions the TA (transient absorption) signal decayed only modestly within the first picosecond, indicating that a substantial fraction of the excited population persisted well beyond the sub nanosecond regime (depicted in Figure 4D). This prolonged excited state residence correlated nicely with the secondary pink fluorescence peak observed in the Video S1, which we have attributed to aggregation induced emission arising from π–π stacking of R‐CDs cores in DMF. In line with the work by Gruebele and coworkers [54] stacking created delocalized excited states that were less efficiently quenched, thereby resulting in an apparently slower decay while enhancing the radiative lifetime.

Conversely, in water the TA response was dramatically faster and decayed more strongly. Excitation at 360 nm yielded a decay with a characteristic time of 0.27 ns (see Figure 4C,E); the residual offset on the nanosecond timescale was very small. The 700 nm probe trace displayed a pronounced plateau between 2 and 4 ps, suggesting a brief bottleneck in energy redistribution—likely the rapid formation of nonradiative pathways such as solvent mediated charge transfer or surface state relaxation. After about 1 ns the residual excited state population was considerably lower than in DMF, consistent with the absence of the second fluorescence band in aqueous media. Attempts to probe the 550 nm excitation in water were hampered by strong Mie scattering from aggregates, precluding reliable kinetic extraction at that wavelength.

Overall, the femtosecond dynamics provided a mechanistic bridge between solvent‐controlled CD aggregation, excited state lifetimes, and the emergence of the additional fluorescence feature in DMF. The π‐π stacked aggregates sustained the excited population for several nanoseconds, whereas isolated R‐CDs in water underwent rapid nonradiative decay, resulting in a markedly weaker long‐lived fluorescence component. These results not only confirmed the AIE hypothesis but also provided the first ultrafast spectroscopic evidence that the aggregation state directly modulates the excited state lifetime of R‐CDs.

### 2.6 Aggregation‐Induced Emission of R‐CDs in Different DMF Concentration

**FIGURE 5:**
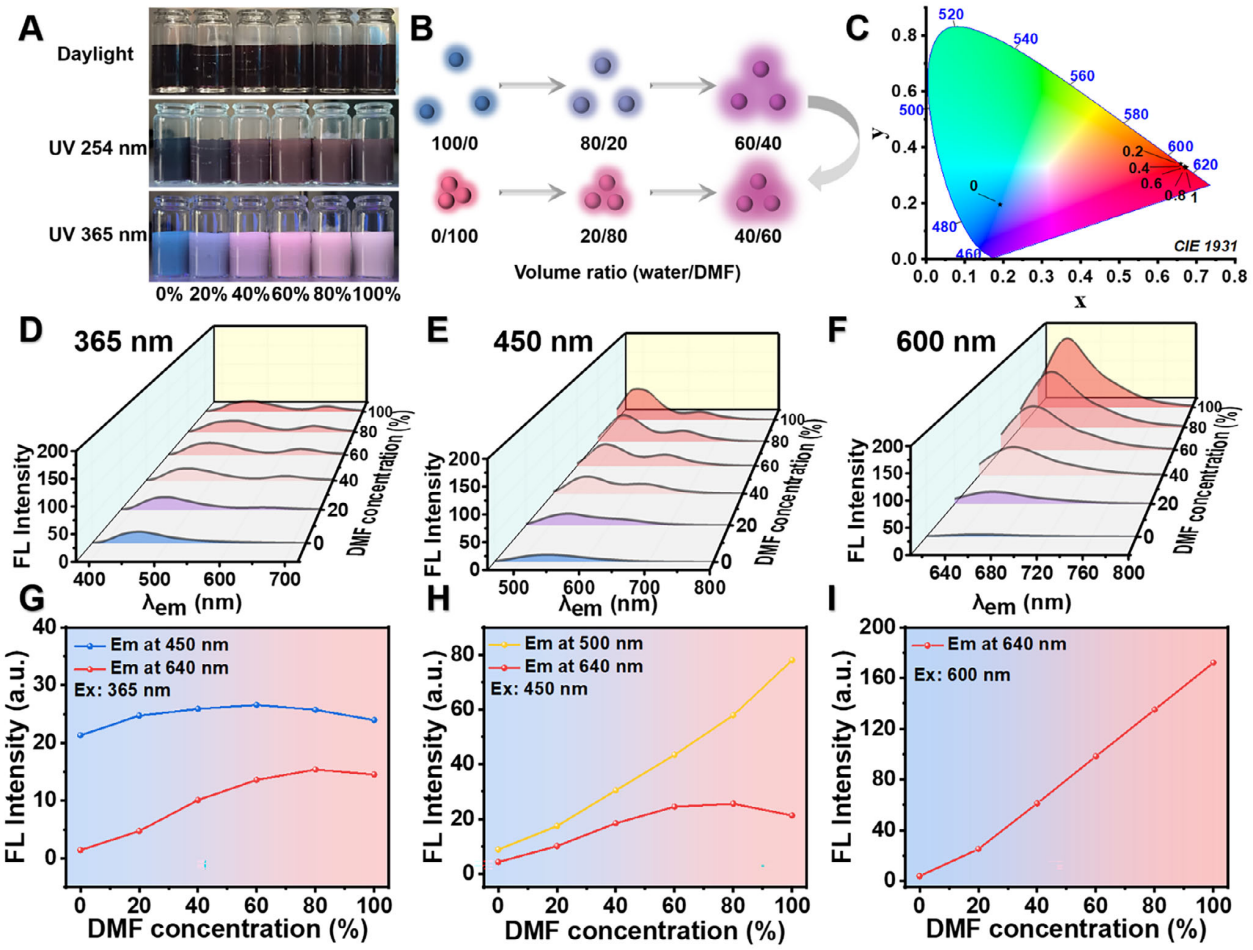
(A) Image of R‐CDs in different DMF concentrations (under daylight, UV 254 nm and UV 365 nm); (B) the aggregation state of R‐CDs in different volume ratios (water/DMF: 100/0, 80/20, 60/40, 40/60, 20/80 and 0/100); (C) chromaticity diagram of R‐CDs in different DMF concentration; (D) (E) (F) FL spectra of R‐CDs in different DMF concentration under different excitation wavelength (365, 450 and 600 nm); (G) intensity change of the emission wavelengths at 450 and 640 nm for R‐CDs in different DMF concentrations at an excitation wavelength of 365 nm; (H) intensity change of emission wavelength at 500 and 640 nm for R‐CDs in different DMF concentrations at an excitation wavelength of 450 nm; (I) intensity change of the emission wavelength at 640 nm for R‐CDs in different DMF concentrations at an excitation wavelength of 600 nm.

As shown in Figure 5A, the FL spectra of R‐CDs (water/DMF) with different DMF concentration were analyzed (R‐CDs concentration: 0.1 mg/mL). As can be seen from the figure, with increasing DMF concentration, under UV irradiation at 365 nm, the color of R‐CDs changed from blue to pink, and the color gradually deepened. The FL spectra of these R‐CDs solutions at excitation wavelengths of 365, 450, and 600 nm were tested (shown in Figure 5D, E,F). As depicted in the figures, with increasing DMF concentration, red fluorescence emission peaks gradually appeared in the 620–650 nm range. From the corresponding fluorescence intensity change curves at specific emission wavelengths, it was observed that, overall, the fluorescence intensity at an emission wavelength of 640 nm showed an upward trend (see Figure 5G, H,I). This indicated that the addition of DMF promoted the emission of red fluorescence from R‐CDs. The color changes were observed using the chromaticity diagram (shown in Figure 5C).

Dynamic light scattering (DLS) was used to measure the particle size of R‐CDs in solutions with 0%, 60%, and 100% DMF concentration (R‐CDs concentration is 0.001 mg/mL). As shown in Figure S7, the average particle size of R‐CDs in pure water was 820.0 nm, in a solution with 60% DMF concentration, the average particle size of R‐CDs reaches 1896.1 nm, and in pure DMF solution, the average particle size of R‐CDs reached 2105.1 nm. It should be noted that the particle size measured by DLS reflects the hydrodynamic diameter (*D*_h_) and not the actual particle sizes of R‐CDs. However, a clear trend was observed: As DMF was added, R‐CDs underwent significant aggregation in solution. This also confirmed that R‐CDs were in a dispersed state in water. And upon addition of DMF, they aggregated, thereby producing bright red fluorescence (depicted in Figure 5B). Therefore, the prepared R‐CDs exhibited a distinct AIE effect.

### 2.7 Aggregation‐Induced Emission of R‐CDs in Different Solvents

**FIGURE 6:**
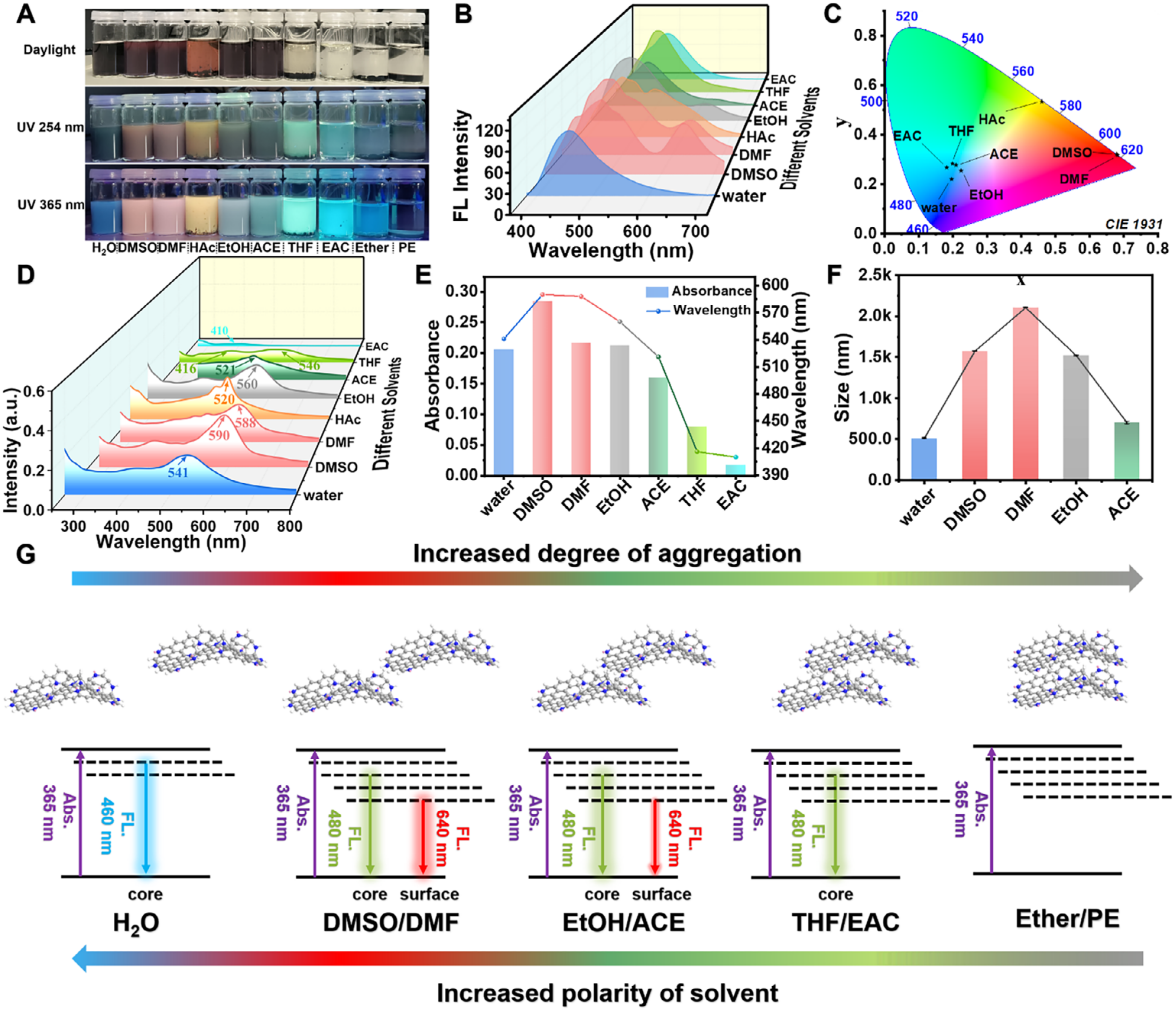
(A) Image of R‐CDs in different solvents (under daylight, UV 254 nm and UV 365 nm); (B) chromaticity diagram of R‐CDs in different solvents; (C) FL spectrum of R‐CDs in different solvents (under excitation wavelength of 365 nm); (D) UV–vis spectrum of R‐CDs in different solvents and (E) optimal absorption wavelength and absorbance; (F) particle size of R‐CDs in different solvents (measurement by DLS); (G) schematic diagram of the aggregation‐induced emission of R‐CDs.

Solubility of R‐CDs in various solvents (water, DMSO, DMF, acetic acid, ethanol, acetone, THF, ethyl acetate, ether, and petroleum ether) was studied at a concentration of 0.1 mg/mL. As shown in Figure 6A, these solvents were arranged in order of decreasing polarity. As can be seen from the figure, R‐CDs were highly soluble in polar solvents, indicating that R‐CDs were also polar. This is because the surface of R‐CDs contained a large number of amino groups, carboxyl groups, and hydroxyl groups present in the raw material. Additionally, we observed that in highly polar acetic acid, R‐CDs were only partially dissolved. This is because the numerous carboxyl groups on the surface of R‐CDs formed competitive hydrogen bonds with the carboxyl groups of acetic acid solvent molecules, preventing complete dissolution of R‐CDs. Additionally, R‐CDs were dissolved more extensively in ethyl acetate than in THF. When R‐CDs were first added to ethyl acetate, they were clearly insoluble. However, ethyl acetate solvent molecules possess carbonyl groups (C═O), which formed strong solvation interactions with the polar groups on the CDs surface (such as ─COOH and ─OH) through dipole‐dipole interactions. Therefore, after 10 to 30 min, R‐CDs dissolved in ethyl acetate. It was also observed that starting from DMSO, as the polarity of the solvent decreased, the fluorescence color of R‐CDs changed from red to yellow, dark green, bright green, blue, and finally colorless and transparent (under UV light of 365 nm). The FL spectra of R‐CDs in each solvent were tested separately under 365 nm excitation (see Figure 6B). As shown in the figure, in aqueous solution, R‐CDs exhibited only a single blue fluorescence emission peak around 450 nm. In DMSO and DMF, R‐CDs exhibited not only a single emission peak around 480 nm but also another peak around 640 nm. Therefore, red fluorescence was observed in these two solvents. Similarly, in acetic acid, although only a small portion of R‐CDs was dissolved, there was a distinct fluorescence emission peak around 540 nm, corresponding to orange‐yellow fluorescence. For ethanol and acetone, there was a slightly elevated fluorescence emission peak around 630 nm, while the green fluorescence emission peak around 470 nm was more prominent. In THF, R‐CDs exhibited a distinct green fluorescence emission peak around 480 nm. In ethyl acetate, R‐CDs also exhibited a fluorescence peak around 480 nm, but a prominent peak appeared around 430 nm, resulting in a green‐blue fluorescence in ethyl acetate.

### 2.8 The Cause of Solvent‐Induced Color Change

The aggregation driver of CDs arose from π‐π stacking induced by their own large conjugated structure. Generally, the interactions between solvent molecules and CDs (such as hydrogen bonds, dipole interactions, and van der Waals forces) competed with the π‐π stacking interactions between CDs. When the interactions between solvent molecules and R‐CDs were stronger than the π‐π interactions between R‐CDs, R‐CDs dispersed uniformly in the solvent (e.g., water), which is why R‐CDs were soluble in water. As polarity decreased, the interactions between solvent molecules and R‐CDs weakened, and π‐π stacking gradually became more dominant, leading to aggregation among R‐CDs. Therefore, in nonpolar solvents, R‐CDs exhibited significant insolubility. Figure 6C shows a chromaticity diagram based on the FL spectra of R‐CDs in different solvents. From the figure, it can be seen that as the polarity of the solvent decreased, the fluorescence wavelength of R‐CDs tended toward the lower fluorescence emission wavelength region.

Because of the π‐π stacking effect, R‐CDs underwent aggregation. Additionally, π‐π interactions caused energy level splitting in R‐CDs, forming lower‐energy bonding orbitals and higher‐energy antibonding orbitals, thereby altering the fluorescence color [55, 56]. There are primarily three types of π‐π stacking: face‐to‐face H‐aggregation, head‐ to‐tail J‐aggregation, and edge‐to‐face T‐aggregation. Among these, H‐aggregation typically caused a blue shift in the absorption spectrum, a decrease in fluorescence intensity, or even quenching, while J‐aggregation resulted in a red shift in the absorption spectrum and an increase in fluorescence intensity [57, 58]. As shown in Figure S6, addition of DMF to the aqueous solution of R‐CDs significantly enhanced the fluorescence and caused a red shift, which was a typical manifestation of J‐aggregation. As shown in FL spectrum Figure 6B, compared to water, R‐CDs solutions in DMF and DMSO exhibited a red‐shifted emission peak at 640 nm. In the UV‐vis spectrum Figure 6D,E, compared to the aqueous solution of R‐CDs, the UV optimal absorption peaks of the DMSO and DMF solutions of R‐CDs also exhibited red‐shifting. These two results also confirmed that R‐CDs undergo J‐aggregation when transitioning from water to DMF and DMSO. Furthermore, Figure 6B depicts that the fluorescence intensity of the emission peak at 640 nm decreased progressively from DMSO, DMF to ethanol, acetone, THF, and ethyl acetate. In Figure 6D,E, it was observed that the optimal absorption peaks of solutions in DMSO, DMF, ethanol, acetone, THF, and ethyl acetate continuously blue‐shifted, and the absorption peak intensity gradually decreased. From this, it was concluded that the decrease in solvent polarity led to a stronger aggregation of R‐CDs, with π‐π stacking transitioning from J‐aggregation to H‐aggregation. This is also why the fluorescence of R‐CDs gradually quenched in ether and petroleum ether. Compared with conventional CDs, the R‐CDs exhibited pronounced solvent‐dependent fluorescence color changes, endowing them with great potential as solvent‐responsive luminescent sensors.

DLS was also used to analyze the particle size of R‐CDs in different solvents (refer to Figure S8). As shown in the Figure 6F, the particle size of R‐CDs increased from water to DMSO and DMF, which was due to the transition of R‐CDs from a dispersed state to an aggregated state in the solvent. In ethanol and acetone, the particle size gradually decreased. This also indicated that the distance between R‐CDs became increasingly shorter, with J‐aggregation transforming into H‐aggregation. In THF and ethyl acetate, the precipitation of R‐CDs was observed visually (shown in Figure S9A). In diethyl ether and petroleum ether, fluorescence quenching even occurred. Figure S9 B,C,D shows the solid powder of R‐CDs and it can be seen when the R‐CDs were completely aggregated, as the fluorescence of R‐CDs quenched completely. As shown in Figure 6G, in water, R‐CDs existed in a dispersed state, with fluorescence emission primarily consisting of blue emission at 450 nm. In DMSO and DMF, R‐CDs exhibited J‐aggregation in a head‐to‐tail way, with red emission at 640 nm being excited, resulting in strong fluorescence. As polarity decreased, π‐π stacking gradually became dominant, and the distance between R‐CDs molecules gradually decreased, slowly transitioning to face‐to‐face H‐aggregation. Therefore, in ethanol and acetone, the intensity of red emission gradually decreased until it disappeared in THF and ethyl acetate. When face‐to‐face H‐aggregation predominated completely, R‐CDs no longer emitted a fluorescence (in ether and petroleum ether). The central role of aggregation in determining fluorescence became clear from the above results. Interestingly, the overall trend of fluorescence color change was also consistent with the typical positive correlation solvatochromism observed in organic dye molecules [59]. Such a fluorescence behavior enables a sensor application based on its predictable color, which can greatly extend the utilization of CDs.

Here, we did not discuss R‐CDs in acetic acid. This is because the high polarity of acetic acid and its high hydrogen bonding donor acidity of 1.12, which is close to that of water (1.17), while the dielectric constant (6.2) and *E_T_* (30) (51.7) of acetic acid were both lower than those of ethanol (refer to, Table S5). Although there was a hydrogen bonding competition between the carboxyl groups of acetic acid molecules and those on the surface of R‐CDs, for the portion of CDs that were soluble in acetic acid, the hydrogen bonds provided by acetic acid molecules to R‐CDs exerted a stronger force. Therefore, the particle size of R‐CDs dissolved in acetic acid (736.0 nm) was close to that of R‐CDs in water (510.0 nm) (see Figure S8F).

### 2.9 Application for Information Coding

Utilizing the fluorescent properties of CDs for information encoding and cryptographic storage is currently one of the most popular applications of CDs [60, 61, 62, 63]. Herein, different information encoding applications were designed by utilizing the photoluminescence and solvent‐induced color change properties of R‐CDs themselves, as well as the gel color change properties of R‐CDs fluorescent hydrogels formed by combining R‐CDs with hydrogels.

### R‐CDs for Information Coding

**FIGURE 7:**
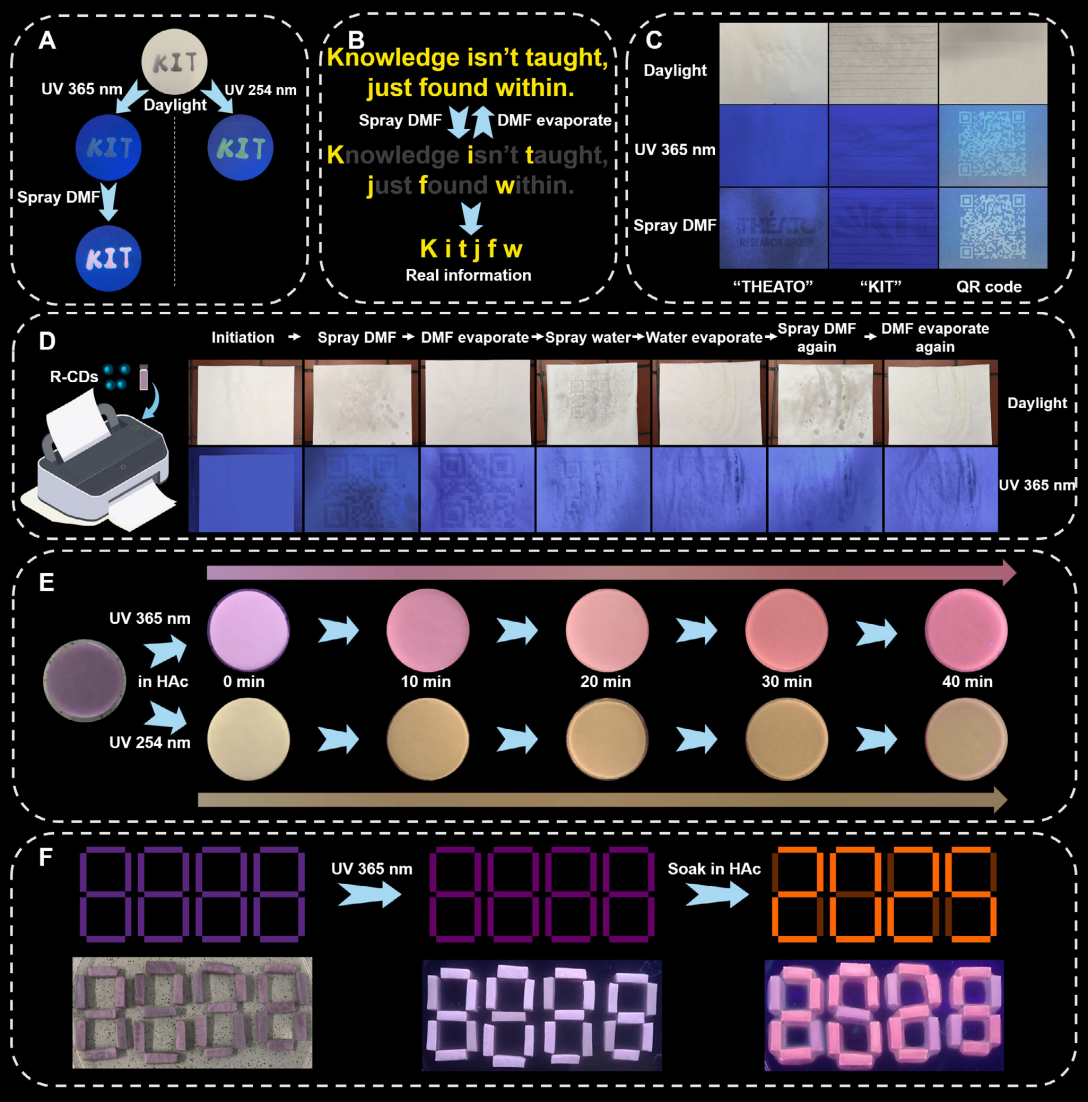
Application of R‐CDs: (A) Color change of writing on filter paper; (B) writing‐based information encoding on filter paper; (C) printing‐based information encoding on different paper substrates (the left is normal A4 paper, the middle is notebook paper, the right is filter paper); (D) process of appearance and disappearance of printed information encoding; (E) photographs of solvent‐responsive fluorescence color change of PVA‐based fluorescent hydrogel under 365 nm excitation; (F) number information coding of PVA‐based fluorescent hydrogels.

First, a simple application study on R‐CDs was conducted. As shown in the Figure 7A and Video S2, the aqueous solution of R‐CDs was loaded into a watercolor pen as an ink to write the purple letters ‘KIT’ on a filter paper. Under 365 nm UV light, ‘KIT’ was visible due to the weak yellow‐green fluorescence, and under 254 nm UV light, it exhibited green fluorescence. When DMF was sprayed onto the text, ‘KIT’ quickly turned into bright red fluorescence. This demonstrated that even on paper, R‐CDs underwent an aggregation‐induced color change. Based on this, the following two information encoding applications were designed.

Information encoding was written on filter paper by using a mixed solution and Rhodamine B (RhB) solution (see Supporting Information). The encoded message was ‘Knowledge isn't taught, just found within.’ As shown in Figure 7B, Figure S10 and Video S3, by controlling the concentration of the fluorescent material, it was observed that under fluorescent light, no information was visible on the filter paper. Under 365 nm irradiation, the message ‘Knowledge wasn't taught, just found within’ became visible. Then, DMF was sprayed onto the paper. Under daylight, no information was visible on the filter paper. However, under 365 nm, the desired content ‘Kitjfw’ became visible. Since DMF is a good solvent for RhB and hence caused RhB molecules to disperse into a single‐molecule state, this resulted in a significant decrease in RhB's fluorescence intensity. However, DMF caused R‐CDs to aggregate and increased their fluorescence intensity. Thus, the true encoded message ‘Kitjfw’ was revealed. When heated, causing the DMF to evaporate, ‘Knowledge isn't taught, just found within’ reappeared. Through this method, the appearance and erasure of encoded information was successfully achieved. This encoding is based on the AIE behavior of CDs developed in this study, differentiating itself from existing other CDs.

The AIE of R‐CDs can also be applied to printing content of information encryption. 0.05 mg/mL R‐CDs aqueous solution was used as ink for an inkjet printer and added to the printer cartridge. Recycled A4 paper, ordinary notebook paper, and filter paper was used as printing paper (refer to Figure 7C). A QR code was printed by using the printer. As shown in Figure 7D and Video S4, without any pretreatment, the QR code was visible under both daylight and UV light. After spraying DMF onto the paper, the QR code gradually appeared. However, the QR code remained invisible under daylight. After evaporation of DMF, the QR code remained visible under UV light. This indicated that the printed QR code was stable. After spraying water on the paper and drying it, the QR code completely disappeared. When DMF was sprayed again, the QR code no longer appeared. This method enabled the appearance and erasure of two‐dimensional information encoding. As shown in Figure 7C, the research group logo, KIT logo, and research group QR code were printed on recycled A4 paper, ordinary notebook paper, and filter paper, respectively. To achieve the effect of no traces being visible under fluorescent light, the concentration of CDs was reduced. Therefore, initially, no traces were visible under 365 nm. After spraying DMF on the paper, the black information content gradually appeared. The QR codes printed on filter paper initially showed a faint blue fluorescence under UV light of 365 nm. After spraying with DMF, the weak blue fluorescence gradually transformed into a brighter white fluorescence.

Furthermore, to evaluate the long‐term stability of R‐CDs printed on paper substrates, multiple copies of a logo were simultaneously printed using the R‐CD ink and subsequently exposed to ambient air under standard laboratory conditions. These printed samples were then individually treated by spraying with DMF after varying exposure durations (specifically on days 1, 2, 5, 7, 9, 14, and 30) (Figure S11). The results demonstrated that even after 30 days of environmental exposure, the printed logo could still be clearly visualized under UV irradiation upon DMF spraying, indicating the exceptional environmental stability and practical robustness of the R‐CDs. Interestingly, the visualized logo gradually blurred and faded within one day post‐activation. This distinct behavior suggested that the R‐CDs possessed a desirable self‐erasing capability after completing the information display, making them highly promising for secure, transient information storage applications (Figure S12).

### Fluorescent Color‐Changing Hydrogels for Information Coding

As shown in the Figure S13, gelatin was used first as the base material for the preparation of fluorescent hydrogels exhibiting a color‐changing behavior. Under 365 nm, the gelatin hydrogels exhibited blue fluorescence. After being immersed in DMF for a period of time, the gelatin hydrogels gradually changed from blue to red. Then, the already red gelatin hydrogels were immersed in water again. Over time, the hydrogels reverted back to a blue fluorescence.

Similarly, PVA was also used as the base material for the preparation of fluorescent color‐changing hydrogels. As shown in Figure 7E, the PVA‐based fluorescent hydrogels were immersed in acetic acid. Under UV light at 365 nm, it was observed that the PVA‐based fluorescent hydrogel underwent a rapid change in fluorescent color within a very short time, quickly transitioning from pink to deep red with a hint of orange.

When comparing the fluorescent color‐changing effects of gelatin and PVA, PVA exhibited a rapid responsiveness in acetic acid. Therefore, we selected PVA‐based hydrogels to design a fluorescent information encoding application. During the study of the optical properties of R‐CDs, we found that when R‐CDs were dissolved in an aqueous solution, their optical properties changed after a period of time. R‐CDs originally exhibited a blue fluorescence in aqueous solutions. Upon addition of DMF, the fluorescence turned red. After the optical properties changed, R‐CDs exhibited a green fluorescence in aqueous solutions, and upon addition of DMF, the fluorescence changed to yellow. The study of this issue indicated that R‐CDs underwent oxidation in aqueous solutions, and the oxidized R‐CDs exhibited this phenomenon (detailed in the Figure S14). Therefore, oxidized R‐CDs were utilized with normal R‐CDs to construct the hydrogel fluorescent information encoding (refer to Figure 7F). Under daylight, a fluorescent hydrogel‐formed number ‘8888’ was visible. Similarly, under 365 nm, pink fluorescence was exhibited, and the number remained ‘8888’. When the hydrogel number was immersed in acetic acid, the PVA‐based fluorescent hydrogels rapidly underwent a color‐changing response. After a few minutes, the actual numerical code ‘2025’ as the secret information became visible.

## 3 Conclusion

In summary, CA and urea were used to synthesize hydrophilic red‐emissive carbon dots (R‐CDs) exhibiting both AIE and ACQ behaviors via a solvothermal method. The R‐CDs were well dispersed in water, showing weak blue fluorescence, whereas the addition of DMF induced their aggregation, leading to bright red emission. The red fluorescence was attributed to the large conjugated π structure of the R‐CDs. The coreaction of citric acid and urea, together with the participation of DMF in the reaction, promoted the formation of pyridinic ***N***, pyrrolic ***N***, and graphitic ***N*** species, thereby facilitating the construction of an extended π‐conjugated system. Owing to this highly conjugated system, R‐CDs were more prone to π–π stacking interactions. Driven by the combined influence of π–π stacking among solute molecules and solvent effects between solute and solvent molecules, the R‐CDs could undergo transformation between J‐type and H‐type aggregation, resulting in solvent‐dependent fluorescence color changes. By the solvent‐responsive fluorescence behavior of R‐CDs, we designed various anticounterfeiting and information encryption applications. Furthermore, by integrating R‐CDs with PVA hydrogels, the solvent‐responsive fluorescent hydrogels capable of rapid fluorescence color switching were fabricated and successfully applied to information encoding. This work not only elucidated the solvent‐dependent emissive behavior of R‐CDs through π–π stacking and the interaction between solvent and solute modulation but also established a general strategy for designing solvent‐responsive luminescent systems. The outstanding solvent sensitivity and optical tunability endowed R‐CDs with great potential for intelligent sensing, anticounterfeiting, and information security applications, providing a versatile platform for the development of next‐generation smart photofunctional materials.

## Author Contributions

**Jiafeng Wan**: Methodology, data curation, investigation, conceptualization, formal analysis, validation, visualization, writing – original draft. **Shiyi Chen**: Methodology, validation, investigation, data curation. **Haopu Su**: Methodology, investigation, validation, data curation. **Tim Muschik**: Methodology, investigation, validation, data curation, writing – original draft, software. **Tongtong Cui**: Methodology, investigation, validation. **Yosuke Akae**: Writing – review and editing. **Dominik Voll**: Writing – review and editing. **Christian W. Schmitt**: Writing – review and editing. **Stefan Bräse**: Methodology, investigation, validation, supervision, writing – review and editing. **Tao Chen**: Methodology, investigation, validation, supervision, writing – review and editing. **Andreas‐Neil Unterreiner**: Methodology, investigation, validation, supervision, writing – review and editing. **Patrick Théato**: Supervision, project administration, funding acquisition, resources, writing – review and editing.

## Funding

Helmholtz Association, China Scholarship Council

## Conflicts of Interest

The authors declare no conflicts of interest.

## Supporting information



Supporting Information is available from the Wiley Online Library or from the author.**Supporting File**: anie73187‐sup‐0001‐SuppMat.docx.

**Supporting File**: anie73187‐sup‐0002‐VideoS.zip.

## Data Availability

The data that support the findings of this study are available from the corresponding author upon reasonable request.
