# Supplementary material for: Uncovering Aggregation‐Induced Emission in Carbon Dots for Color‐Changing Hydrogels and Information Encryption
Source: Angew Chem Int Ed Engl. 2026 Jun 13;65(36):e7943243. doi: 10.1002/anie.7943243 (PMC13528517; doi:10.1002/anie.7943243)
Supplement: Supplementary file 1 [file ANIE-65-e7943243-s002.docx]

Supporting Information

**Uncovering Aggregation-Induced Emission in Carbon Dots for Color-Changing Hydrogels and Information Encryption**

Jiafeng Wan, Shiyi Chen, Haopu Su, Tim Muschik, Tongtong Cui, Yosuke Akae, Dominik Voll, Christian W. Schmitt, Stefan Bräse, Tao Chen, Andreas-Neil Unterreiner, Patrick Théato*

J. Wan, S. Chen, T. Cui, T. Muschik, Dr. Y. Akae, Dr. D. Voll, Prof. Dr. P. Théato
Institute for Chemical Technology and Polymer Chemistry (ITCP)
Karlsruhe Institute of Technology (KIT)
Engesserstr. 18, 76131 Karlsruhe, Germany
E-mail: [patrick.theato@kit.edu](mailto:patrick.theato@kit.edu)

H. Su, Prof. Dr. S. Bräse
Institute of Organic Chemistry (IOC)
Karlsruhe Institute of Technology (KIT)
Kaiserstraße 12, 76131 Karlsruhe, Germany

T. Muschik, Prof. Dr. A.-N. Unterreiner
Institute of Physical Chemistry (IPC)
Karlsruhe Institute of Technology (KIT)
Kaiserstraße 12, 76131 Karlsruhe

Dr. Y. Akae
Research Fellow of Japan Society for the Promotion of Science
102-0083 Tokyo, Japan

Dr. C. W. Schmitt, Prof. Dr. P. Théato
Soft Matter Synthesis Laboratory - Institute for Biological Interfaces III (IBG-3)
Karlsruhe Institute of Technology (KIT)
76344 Eggenstein-Leopoldshafen, Germany

Prof. Dr. S. Bräse
Institute of Biological and Chemical Systems – Functional Molecular Systems (IBCS-FMS)
Karlsruhe Institute of Technology (KIT)
Kaiserstraße 12, 76131 Karlsruhe, Germany

Prof. Dr. T. Chen
State Key Laboratory of Advanced Marine Materials
Zhejiang Key Laboratory of Extreme-environmental Material Surfaces and Interfaces
Ningbo Institute of Materials Technology and Engineering
Chinese Academy of Sciences
Ningbo 315201, China

Prof. Dr. T. Chen
School of Chemical Sciences
University of Chinese Academy of Sciences
Beijing 100049, China

**Table of Contents**

**1.	Experimental Section**	**3**

1.1	Materials and reagents	3

1.2	Synthesis of B-CDs, G-CDs, R-CDs.	3

1.3	Synthesis of CDs-1, CDs-2, CDs-3, CDs-4	4

1.4	Oxidation study of R-CDs	5

1.5	Writing-based information encoding on filter paper	5

1.6	Information-encoded printing	5

1.7	Preparation of fluorescent hydrogels	6

1.8	Fabrication of fluorescent hydrogel patterns (digits)	7

1.9	Characterization techniques	7

**2.	Result and discussion**	**9**

Figure S1.	9

Table S1.	10

Table S2.	11

Table S3.	12

Table S4.	13

Figure S2.	14

Figure S3.	15

Figure S4.	16

Figure S5.	17

Figure S6.	18

Figure S7.	19

Figure S8.	20

Table S5.	21

Figure S9.	22

Figure S10.	23

Figure S11.	24

Figure S12.	25

Figure S13.	26

**3.	Oxidized R-CDs**	**27**

Figure S14.	28

**References**	**29**

1. Experimental Section
   1.1 Materials and reagents

Citric acid (CA, >98.0%, TCI Chemicals), urea (> 99.5%, Sigma Aldrich), *N*,*N*-Dimethylformamide (DMF, assay 100%, VWR), Ethanol (>99.5%, Sigma Aldrich), L-(+)-Ascorbic acid (Vitamin C, >98%, Alfa Aesar), NaS_2_O_3_ (Thermo scientific), Rhodamine B (RhB, >98.0%, TCI Chemicals), polyvinyl alcohol (PVA, *M*_w_ = 89000-98000, >99%, Sigma Aldrich), gelatin (Type A, Sigma Aldrich).

1.2 Synthesis of B-CDs, G-CDs, R-CDs

**Synthesis of R-CDs:** 1 g of CA (about 5.2 mmol) and 2 g (about 33.3 mmol) of urea were added to 20 mL of DMF. After complete dissolution. The solution was transferred into a 50 mL hydrothermal reactor lined with poly(*p*-phenylene). The reactor was placed in a forced-air drying oven at 160 °C for 10 h. After completion of the reaction, the reactor was naturally cooled to room temperature to obtain the crude R-CDs with red fluorescence. The crude product was filtered through a 0.2 μm polyethersulfone membrane to remove large particles. Subsequently, the filtrate was dialyzed against deionized water using a 3000 Da dialysis membrane for 24 h in the dark, with the water refreshed every 8 h. After dialysis, a dark R-CDs aqueous solution was obtained. Finally, the solution was freeze dried and yield black R-CDs powder (about 0.382 g, yield :12.7%).

**Synthesis of G-CDs:** 1 g of CA (about 5.2 mmol) and 2 g (about 33.3 mmol) of urea were added to 20 mL of ethanol. After complete dissolution. The solution was transferred into a 50 mL hydrothermal reactor lined with poly(*p*-phenylene). The reactor was placed in a forced-air drying oven at 160 °C for 10 h. After completion of the reaction, the reactor was naturally cooled to room temperature to obtain the crude G-CDs with green fluorescence. An excess amount of acetone was then added into the filtrate, followed by thorough stirring and centrifugation at 4000 r/min. The centrifugation was repeated several times until no further precipitate was observed. The final precipitate was redissolved in deionized water and freeze dried to obtain G-CDs powder (about 0.324 g, yield :10.8%).

**Synthesis of B-CDs:** 1 g of CA (about 5.2 mmol) and 2 g (about 33.3 mmol) of urea were added to 20 mL of deionized water. After complete dissolution. The solution was transferred into a 50 mL hydrothermal reactor lined with poly(*p*-phenylene). The reactor was placed in a forced-air drying oven at 160 °C for 10 h. After completion of the reaction, the reactor was naturally cooled to room temperature to obtain the crude B-CDs with blue fluorescence. The crude product was filtered through a 0.2 μm polyethersulfone membrane to remove large particles. Subsequently, the filtrate was dialyzed against deionized water using a 3000 Da dialysis membrane for 24 h in the dark, with the water refreshed every 8 h. After dialysis, B-CDs aqueous solution was obtained. Finally, the solution was freeze dried and yield B-CDs powder (about 0.216 g, yield :7.2%).

1.3 Synthesis of CDs-1, CDs-2, CDs-3, CDs-4

**Synthesis of CDs-1 solution:** 1 g of CA (about 5.2 mmol) was added to 20 mL DMF. After complete dissolution. The solution was transferred into a 50 mL hydrothermal reactor lined with poly(*p*-phenylene). The reactor was placed in a forced-air drying oven at 160 °C for 10 h. After completion of the reaction, the reactor was naturally cooled to room temperature to obtain the crude CDs-1 with blue fluorescence. The crude product was filtered through a 0.2 μm polyethersulfone membrane to remove large particles to obtain a CDs-1 solution.

**Synthesis of CDs-2 solution:** 2 g of urea (about 33.3 mmol) was added to 20 mL DMF. After complete dissolution. The solution was transferred into a 50 mL hydrothermal reactor lined with poly(*p*-phenylene). The reactor was placed in a forced-air drying oven at 160 °C for 10 h. After completion of the reaction, the reactor was naturally cooled to room temperature to obtain the crude CDs-2 with orange fluorescence. The crude product was filtered through a 0.2 μm polyethersulfone membrane to remove large particles to obtain a CDs-2 solution.

**Synthesis of CDs-3 solution:** 1 g of CA (about 5.2 mmol) was dissolved in 20 mL DMF, and the solution was transferred into a 50 mL hydrothermal reactor lined with a poly(*p*-phenylene). The reactor was placed in a forced-air drying oven at 160 °C for 5 h. After cooling to room temperature, a CDs solution with blue fluorescence was obtained. Subsequently, 2 g of urea was added into the solution and stirred until completely dissolved. The resulting mixture was again transferred into the reactor and heated at 160 °C for another 5 h. The final product was filtered through a 0.2 μm polyethersulfone membrane to obtain a CDs-3 solution.

**Synthesis of CDs-4 solution:** 1 g of CA (about 5.2 mmol) was dissolved in 20 mL DMF, and the solution was transferred into a 50 mL hydrothermal reactor lined with a poly(*p*-phenylene). The reactor was placed in a forced-air drying oven at 160 °C for 5 h. After cooling to room temperature, a CDs solution with blue fluorescence was obtained. The crude product was filtered through a 0.2 μm polyethersulfone membrane to remove large particles, followed by dialysis against deionized water using a 3000 Da dialysis membrane for 24 h in the dark, with the water renewed every 8 h. After dialysis, the solution was freeze dried to obtain the preliminary CDs. The obtained CDs were then mixed with 2 g of urea, dissolved in 20 mL of DMF, and transferred into the autoclave again. The mixture was heated at 160 °C for 10 h in a forced-air oven. The final product was filtered through a 0.2 μm polyethersulfone membrane to obtain a CDs-4 solution.

1.4 Oxidation study of R-CDs

A 1 mg/mL aqueous solution of R-CDs was prepared. Four sample vials were taken and labeled as 1, 2, 3, and 4. Each vial contained 5 mL of the R-CDs solution.

Group 1: No treatment was applied. The vial cap was left open, allowing exposure to air.

Group 2: The vial was tightly capped and sealed with parafilm to prevent air from entering.

Group 3: 4 mg of solid powder vitamin C was added, and the vial was capped.

Group 4: 7 mg of solid Na₂S₂O₃ was added, and the vial was capped.

After 5 days, a small amount of solution from each group was mixed with DMF, and their fluorescent color changes were tested.

1.5 Writing-based information encoding on filter paper

First, aqueous solutions of R-CDs (0.05 mg/mL) and RhB (0.0005 mg/mL) were prepared. The two solutions were mixed at a volume ratio of 4:1 to obtain a fluorescent ink mixture. The information content was “Knowledge isn’t taught, just found within”. The letters “K”, “i”, “t”, “j”, “f”, and “w” were written using the mixed solution, while the remaining parts were written using the 0.0005 mg/mL RhB solution.

1.6 Information-encoded printing

A 0.05 mg/mL aqueous solution of R-CDs was prepared. Empty printer cartridges were rinsed with ethanol to remove the original ink. After the ethanol had completely evaporated, the cartridges were rinsed with deionized water to ensure cleanliness. Then, 5 mL of the R-CDs solution was added to the cartridges. Blow air was applied to the ink inlet using a blow bottle until a small amount of R-CDs solution dripped from the bottom. The desired content was printed using the corresponding printing program on a computer (type of printer: HP Deskjet 2050 j510, Hewlett-Packard, Palo Alto, CA, USA).

1.7 Preparation of fluorescent hydrogels

**Preparation of** **gelatin-based fluorescent hydrogels:** 2 g of gelatin was weighed using weighing paper. 9 mL of deionized water was added into a 20 mL beaker and heated in a water bath at 60 °C. After the temperature stabilized, the gelatin was added to hot water and stirred with a magnetic stirrer at 400 r/min until completely dissolved. The aqueous solutions of R-CDs (1 mg/mL) and a Na₂S₂O₃ solution (500 µL/mg) were prepared, separately. Then, 1 mL of the R-CDs solution and 80 µL of the Na₂S₂O₃ solution were mixed thoroughly and added into the gelatin solution. After stirring for 15 min, the mixture was transferred into molds and cooled at 4 °C to form hydrogels.

**Preparation of** **PVA-based fluorescent hydrogels:** 1.4 g of PVA powder was weighed using weighing paper. 7 mL of deionized water was added into a 20 mL beaker and heated in a water bath at 85 °C. After the temperature stabilized, the PVA was added into the hot water and stirred with a magnetic stirrer at 400 r/min until completely dissolved. The aqueous solutions of R-CDs (1 mg/mL) and a Na₂S₂O₃ solution (500 µL/mg) were prepared, separately. Then, 1 mL of the R-CDs solution and 80 µL of the Na₂S₂O₃ solution were mixed thoroughly. Separately, 0.2 g of BA was dissolved in 2 mL of deionized water at 75 °C. The BA solution was mixed with the R-CDs solution, and the mixture was subsequently added into the PVA solution. After stirring for 5 ~ 10 min, the mixture was poured into molds and reacted in an oven at 50 °C for 4 h. The pre-gels were then subjected to a freeze-thaw cycle by freezing at -20 °C for 12 h and thawing at room temperature for 2 h. This cycle was repeated three times to obtain the final PVA-based fluorescent hydrogels.

**Preparation of PVA-based oxidized R-CDs fluorescent hydrogels:** 1.4 g of PVA powder was weighed using weighing paper. 7 mL of deionized water was added into a 20 mL beaker and heated in a water bath at 85 °C. After the temperature stabilized, the PVA was added into the hot water and stirred with a magnetic stirrer at 400 r/min until completely dissolved. The aqueous solutions of oxidized R-CDs (1 mg/mL) were prepared. Separately, 0.2 g of BA was dissolved in 2 mL of deionized water at 75 °C. The BA solution was mixed with the 1 mL of oxidized R-CDs solution, and the mixture was subsequently added into the PVA solution. After stirring for 5 ~ 10 min, the mixture was poured into molds and reacted in an oven at 50 °C for 4 h. The pre-gels were then subjected to a freeze-thaw cycle by freezing at -20 °C for 12 h and thawing at room temperature for 2 h. This cycle was repeated three times to obtain the final PVA-based fluorescent hydrogels.

1.8 Fabrication of fluorescent hydrogel patterns (digits)

Watch dishes with a diameter of 110 mm were used as molds to prepare PVA-based fluorescent hydrogels and PVA-based oxidized R-CDs fluorescent hydrogels. After preparation, the hydrogels were cut into rectangular strips with dimensions of 5 mm × 15 mm. In total, 21 strips of PVA-based fluorescent hydrogels and 7 strips of PVA-based oxidized R-CDs fluorescent hydrogels were obtained. The PVA-based fluorescent hydrogel strips were assembled to construct the “2025” portion of the pattern, while the PVA-based oxidized R-CDs fluorescent hydrogel strips were used to assemble the remaining segments. Together, these components yielded a final design in the shape of the digits “8888”.

1.9 Characterization techniques

The high-resolution transmission electron microscope (HRTEM) model used is FEI Tecnai G2 F20 (Hillboro, OR, USA). Attenuated total reflection Fourier transform infrared (ATRFTIR) spectra were recorded on a Vertex 80 from Bruker (Billerica, Massachusetts, USA). Nuclear magnetic resonance (NMR) spectra were recorded on a BRUKER Avance 500 NMR instrument at 500 MHz for 1H NMR, and 126 MHz for 13C NMR. X-ray photoelectron spectroscopy (XPS) analysis was obtained using a Thermo Scientific K-Alpha (Thermo Fisher Scientific, USA). All high-resolution XPS spectra were calibrated using the adventitious carbon C 1s peak (284.8 eV) as an internal standard. To distinguish the intrinsic carbon backbone, the C 1s spectra were carefully deconvoluted by anchoring the C=C and C–C components at 284.4 eV and 284.8 eV, respectively. To minimize fitting redundancy and preserve physical validity, the line shapes of these two adjacent components were strictly coupled, sharing identical full-width at half-maximum (FWHM) values and Gaussian-Lorentzian mixing ratios.^[1-2]^ UV-Vis absorption spectra were recorded using an Agilent Cary3500 Multizonen-Peltier-UV-Vis (Figure 3A) and Lambda 650 spectrophotometer (PerkinElmer, USA) (Figure 6D). Fluorescence spectra were obtained using a Cary Eclipse Fluorescence Spectrophotometer (Varian, Inc., Palo Alto, CA, USA). DLS measurements were conducted at a scattering angle of 90° using a light scattering setup equipped with an ALV-SP125 goniometer, an ALV/LSE5004 multi tau correlator, a fiber optical ALV/High QE APD avalanche photodiode with pseudo-cross correlation (ALV Laservertriebsgesellschaft mbH, Langen, Germany). The DLS measurements are analyzed using two modes: GAUSSIAN distribution and NICOMP distribution. Both modes can be used to describe the size of R-CDs in solution. In the figures, the results with the smallest measurement error are selected as the basis for analysis. Transient absorption spectra with sub-100 fs temporal resolution were acquired using a commercial femtosecond laser system (Astrella, Coherent; 1 kHz, 35 fs, 7 μJ) coupled to a TOPAS optical parametric amplifier, providing tunability of the pump wavelength across the UV–Vis–NIR range. Probe pulses were generated in a rotating CaF₂ optical window, covering 350–700 nm. Detection employed two CCD cameras (Entwicklungsbüro Stresing) for simultaneous acquisition of probe intensities before (I₀) and after (I) the sample, enabling calculation of the pump-induced change in optical density (ΔOD). The sample was micro-stirred to prevent local heating and photodegradation. Further details of the setup are provided elsewhere.^[3]^ Data was recorded using an in-house written LabView program and processed with KiMoPack.^[4]^

2. Result and discussion

**Figure S1.** Schematic illustration of the preparation of multicolor CDs.

**Table S1.** The elemental content of multicolor CDs.

|  | C 1s | N 1s | O 1s |
| --- | --- | --- | --- |
| B-CDs | 53.61% | 13.40% | 32.99% |
| G-CDs | 59.20% | 17.10% | 23.70% |
| R-CDs | 60.67% | 18.52% | 20.81% |

**Table S2.** Chemical bond content of high-resolution C 1s of multicolor CDs.

|  | C-C | C=C | C-O/C-N | C=O/C=N | -COOH |
| --- | --- | --- | --- | --- | --- |
| B-CDs | 31.25% | 18.49% | 24.05% | 11.81% | 14.40% |
| G-CDs | 23.96% | 28.03% | 28.33% | 13.68% | 6.10% |
| R-CDs | 18.68% | 30.00% | 32.17% | 10.24% | 8.9% |

**Table S3.** Chemical bond content of high-resolution N 1s of multicolor CDs.

|  | pyridinic ***N*** | pyrrolic ***N*** | graphitic ***N*** |
| --- | --- | --- | --- |
| B-CDs | 15.01% | 43.63% | 41.36% |
| G-CDs | 29.58% | 55.86% | 14.56% |
| R-CDs | 28.51% | 54.17% | 17.32% |

**Table S4.** Chemical bond content of high-resolution O 1s of multicolor CDs.

|  | C=O | C-O |
| --- | --- | --- |
| B-CDs | 73.52% | 26.48% |
| G-CDs | 91.93% | 8.07% |
| R-CDs | 94.87% | 5.13% |

**Figure S2.** Schematic illustration of the preparation of (A) CDs-1, (B) CDs-2, (C) CDs-3, (D) CDs-4.

**Figure S3.** FL spectra of CDs-1: (A) summary, (B) excitation wavelength: 260 nm ~ 430 nm, (C) excitation wavelength: 440 nm ~ 600 nm; FL spectra of CDs-2: (D) summary, (E) excitation wavelength: 260 nm ~ 490 nm, (F) excitation wavelength: 500 nm ~ 600 nm; FL spectra of CDs-3: (G) summary, (H) excitation wavelength: 260 nm ~ 490 nm, (I) excitation wavelength: 500 nm ~ 600 nm; FL spectra of CDs-4: (J) summary, (K) excitation wavelength: 260 nm ~ 490 nm, (L) excitation wavelength: 500 nm ~ 610 nm.

**Figure S4.** (A) image of mixture of CDs-1 and CDs-2; FL spectra of mixture of CDs-1 and CDs-2: (B) summary, (C) excitation wavelength: 260 nm ~ 430 nm, (D) excitation wavelength: 440 nm ~ 600 nm; (E) image of R-CDs; (F) FL spectrum of R-CDs.

**Figure** **S5**. Reversible fluorescence switching of R-CDs over three consecutive cycles.

**Figure S6.** The left is an image of R-CDs aqueous solution and their FL spectra; The middle is an image of R-CDs aqueous solution after adding DMF and their FL spectra; The right is an image of R-CDs DMF solution and their FL spectra.

**Figure S7.** The size of R-CDs in different DMF content: (A) 100% water/0% DMF; (B) 40% water/60%DMF; (C) 0% water/ 100% DMF. (Measurements by DLS. The DLS measurements are analyzed using two modes: GAUSSIAN distribution and NICOMP distribution. Both modes can be used to describe the size of R-CDs in solution. In the figures, the results with the smallest measurement error are selected as the basis for analysis.)

**Figure S8.** The size of R-CDs in different solvents: (A) water; (B) DMSO; (C) DMF; (D) ethanol; (E) acetone; (F) acetic acid. (Measurements by DLS. The DLS measurements are analyzed using two modes: GAUSSIAN distribution and NICOMP distribution. Both modes can be used to describe the size of R-CDs in solution. In the figures, the results with the smallest measurement error are selected as the basis for analysis.)

**Table S5.** Comparative table of polarity parameters.^[5-7]^

| Solvent | *Dielectric constant*/ε | *E_T_*(30) (kcal/mol) | *Hydrogen Bond Donor* *Acidity*/α |
| --- | --- | --- | --- |
| H_2_O | 78.4 | 63.1 | 1.17 |
| DMSO | 46.7 | 45.1 | 0 |
| DMF | 36.7 | 43.2 | 0 |
| HAc | 6.2 | 51.7 | 1.12 |
| EtOH | 24.5 | 51.9 | 0.83 |
| ACE | 20.7 | 42.2 | 0.08 |
| THF | 7.5 | 37.4 | 0 |
| EAC | 6.0 | 38.1 | 0 |

Note

*E_T_*(30): Molar Electronic Transition Energy, which specifically refers to the electronic transition energy of the so-called Reichardt's dye (a pyridinium-*N*-phenolate betaine) in different solvents.^[5]^

**Figure S9.** (A) The image of R-CDs dissolved in THF (left) and EAC (right); The image of R-CDs powder under daylight (B), UV 254 nm (C) and UV 365 nm (D).

**Figure S10.** R-CDs applied for writing-based information encoding on filter paper.

**Figure S11**. Stability demonstration of the R-CDs. (Visualization results of identically printed information upon DMF spraying after various exposure periods. Top: before DMF spraying; Bottom: after DMF spraying)

**Figure S12**. Sustainability of information transfer after DMF spraying.

**Figure S13.** Photographs of solvent-responsive fluorescence color change of gelatin-based fluorescent hydrogels under 365 nm excitation.

3. Oxidized R-CDs

During the study of the optical properties of R-CDs, it was observed that the as-prepared R-CDs exhibited blue fluorescence in aqueous solution, while the addition of DMF induced red fluorescence. After a period of time, the optical properties of R-CDs in aqueous solution underwent noticeable changes: the R-CDs displayed green fluorescence in water, and upon the addition of DMF, they exhibited orange-yellow fluorescence. We speculate that R-CDs are gradually oxidized in water, leading to modifications of their surface functional groups, which in turn alter the defect states and consequently the optical properties. To verify this phenomenon, a series of oxidation experiments were designed.

From the **Video S5**, it was observed that in experimental group 1, the untreated R-CDs showed changes in fluorescence after 5 days. In experimental group 2, R-CDs subjected to physical anti-oxidation exhibited no significant change in their fluorescence properties, maintaining blue fluorescence in water and red fluorescence upon the addition of DMF. Similarly, the fluorescence properties of R-CDs in experimental groups 3 and 4, which were treated with antioxidants, remained consistent with the initial R-CDs.

Based on these observations, IR and NMR analyses were used to investigate the structure of oxidized R-CDs (OR-CDs), and comparisons were made with the original R-CDs (**Figure S12**). **Figure S12 A** showed that, compared with R-CDs, the OR-CDs exhibited a significant increase in the C=O peak around 1650 cm⁻¹, indicating that the OR-CDs were likely oxidized. **Figure S12 B** indicate that the peak at ~3.0 ppm likely corresponds to an amide proton (-N(CH_3_)-CO-), and this peak exhibits significantly reduced signal intensity in OR-CDs. The peak at ~7.9 ppm likely represents an aromatic proton from pyridine ring or pyrrole ring. The diminished signal intensity in OR-CDs suggests disruption of the aromatic system, thereby affecting the photoluminescence properties of OR-CDs.

**Figure S14** (A) IR of OR-CDs and R-CDs; (B) ^1^H NMR of OR-CDs and R-CDs (solvent: D_2_O, 500 MHz, 25 ℃).

References

[1]	J. Díaz, G. Paolicelli, S. Ferrer, F. Comin, *Physical Review B* **1996**, *54*, 8064-8069, <https://doi.org/10.1103/PhysRevB.54.8064>.

[2]	R. Haerle, E. Riedo, A. Pasquarello, A. Baldereschi, *Physical Review B* **2001**, *65*, 045101, <https://doi.org/10.1103/PhysRevB.65.045101>.

[3]	J. O. Wenzel, J. Werner, A. Allgaier, J. van Slageren, I. Fernández, A.-N. Unterreiner, F. Breher, *Angewandte Chemie International Edition* **2024**, *63*, e202402885, <https://doi.org/10.1002/anie.202402885>.

[4]	C. Müller, T. Pascher, A. Eriksson, P. Chabera, J. Uhlig, *The Journal of Physical Chemistry A* **2022**, *126*, 4087-4099, <https://doi.org/10.1021/acs.jpca.2c00907>.

[5]	C. Reichardt, *Chemical Reviews* **1994**, *94*, 2319-2358, <https://doi.org/10.1021/cr00032a005>.

[6]	M. J. Kamlet, J. L. M. Abboud, M. H. Abraham, R. W. Taft, *The Journal of Organic Chemistry* **1983**, *48*, 2877-2887, <https://doi.org/10.1021/jo00165a018>.

[7]	A. A. Maryott, E. R. Smith, *Table of dielectric constants of pure liquids, Vol. 514*, US Government Printing Office, **1951**.
